# Small RNA-mediated genetic switches coordinate ALG-3/4 small RNA pathway function

**DOI:** 10.1093/nar/gkae586

**Published:** 2024-07-05

**Authors:** Trilotma Sen, Cara McCormick, Alicia K Rogers

**Affiliations:** Department of Biology, University of Texas at Arlington, Arlington, TX 76019, USA; Department of Biology, University of Texas at Arlington, Arlington, TX 76019, USA; Department of Biology, University of Texas at Arlington, Arlington, TX 76019, USA

## Abstract

Coordination of gene regulatory networks is necessary for proper execution of cellular programs throughout development. RNA interference (RNAi) is an essential regulatory mechanism in all metazoans. Proper RNAi-mediated gene regulation requires coordination of several RNAi branches to ensure homeostasis. For example, in *Caenorhabditis elegans*, the Argonautes, ALG-3 and ALG-4, are expressed specifically during spermatogenesis (L4 stage) and bind small interfering RNAs (siRNAs) complementary to sperm-enriched genes. We find that *alg-3* and *alg-4* are regulated by siRNAs. Our work shows that gene switches are operated via these siRNAs to regulate the Argonautes’ expression in a temporal manner. This RNAi-to-RNAi regulatory cascade is essential for coordinating ALG-3/4 pathway function, particularly during heat stress, to provide thermotolerant sperm-based fertility. This work provides insight into one regulatory motif used to maintain RNAi homeostasis, across developmental stages, despite environmental stressors. As RNAi pathways are evolutionarily conserved, other species likely use similar regulatory architectures to maintain RNAi homeostasis.

## Graphical Abstract

**Graphical Abstract:**
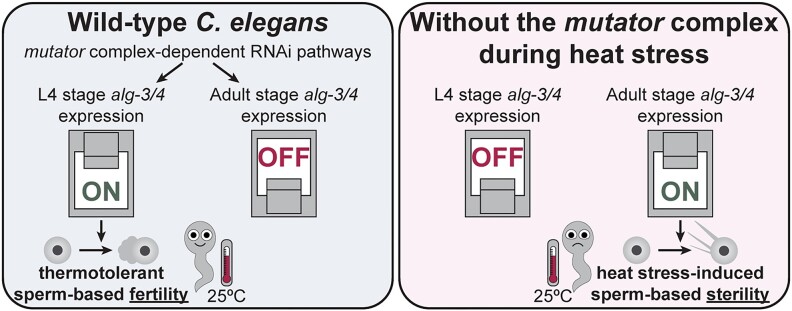

## Introduction

Perturbations to gene regulatory networks caused by routine environmental or genetic stressors can have catastrophic effects on physiological processes, such as fertility. In hermaphroditic *Caenorhabditis elegans*, the same germline tissue sequentially completes spermatogenesis and oogenesis during discrete windows of developmental time (the last larval stage (L4) and adult stage, respectively) (1). While spermatogenesis and oogenesis are temporally separated, sperm and oocytes are derived from mitotically replicating nuclei at the distal tip of the gonadal arms, which undergo meiosis as they progress through the germline towards the proximal end of the gonad (2). Thus, proper coordination of the gene regulatory networks that govern the switch from spermatogenesis to oogenesis within the *C. elegans* germline is essential for determining germ cell fate and maintaining the animal's oocyte- and sperm-based fertility (3–16).

RNA interference (RNAi) is an evolutionarily conserved gene regulatory mechanism responsible for coordinating endogenous gene expression in a temporal and cell specific manner throughout development in all animals. Furthermore, RNAi pathways safeguard the germline genome's integrity from transposons and protect germ cell identity through maintenance of chromatin states. Thus, RNAi plays a crucial role in fertility. Key components of RNAi pathways are RNA-induced silencing complexes (RISC), which are made up of an Argonaute (AGO) protein and its associated small RNA. The small RNAs, typically ∼20–30 nucleotides (nt) in length, guide AGOs to target transcripts to initiate either transcriptional or post-transcriptional silencing (17–23). There are three main classes of small RNAs – microRNAs, small-interfering RNAs (siRNAs), and Piwi-interacting RNAs (piRNAs). In *C. elegans*, two populations of siRNAs, 26G-RNAs and 22G-RNAs, are categorized based on their size, mechanism of biogenesis, and AGO co-factors. 26G-RNAs are dependent on Dicer and the enhancer of RNAi (*eri*) class of genes for their biogenesis and are 26 nt in length with a 5′ guanine (G) (24–26). These 26G-RNAs form complexes with specific AGOs to form ‘primary’ RISC that recognize target transcripts (27,28). Recognition by primary RISC then triggers RNA-dependent RNA polymerase (RdRP)-mediated amplification of ‘secondary’ siRNAs from the target transcript. These secondary siRNAs are 22 nt in length with a 5′ G (22G-RNAs) and can be complexed into ‘secondary’ RISC. Amplification of 22G-RNAs is critical for maintaining robust target regulation and can occur in both a *mutator* complex-dependent or independent manner. The *mutator* complex is nucleated around the intrinsically disordered protein, MUT-16, and is housed within perinuclear germ granules called *Mutator* foci, which sit adjacent to P granules, the perinuclear hubs for RISC-mediated mRNA surveillance (17,18,21,22,29).

There are several distinct branches of RNAi whose components localize to P granules (ALG-3/4, CSR-1, ERGO-1, PRG-1 and RDE-1). Each RNAi pathway branch is defined by the primary and secondary RISC it uses, their mechanism of action on targets, and the branch's dependency on the *mutator* complex for production of 22G-RNAs. The ERGO-1, PRG-1 and RDE-1 RNAi branches use *mutator* complex-dependent 22G-RNA amplification, whereas the ALG-3/4 and CSR-1 branches utilize the RdRP, EGO-1, to achieve *mutator*-independent 22G-RNA amplification (29–31). Each RNAi branch regulates a distinct set of target genes. For example, the ALG-3/4 and CSR-1 pathways have been shown to be essential for balancing RNAi-mediated positive and negative regulation to control appropriate expression levels of spermatogenesis-enriched genes during the narrow timeframe of the L4 developmental stage (24,25,30,32–38). Maintenance of homeostasis between the distinct, yet interdependent, RNAi pathway branches is critical for appropriate gene regulation, however, many questions remain about the molecular mechanisms that ensure RNAi homeostasis.

Mutations in factors of the different RNAi branches can lead to a temperature-sensitive loss of fertility, termed the mortal germline phenotype (*mrt*), that manifests at differing generations (17,25,29,39–42). When *C. elegans* are grown at elevated temperature, the undifferentiated germ cells that give rise to mature sperm and oocytes are constitutively exposed to heat stress during the transition from the L4 stage and adult stage, thus *mrt* phenotypes can be caused by loss of oocyte-based fertility, loss of sperm-based fertility, or both. For example, animals with a null mutation in the core component of the *mutator* complex, MUT-16, exhibit both oocyte- and sperm-based *mrt* phenotypes when exposed to heat stress (31,39). The oogenesis-based effect of *mut-16* manifests after three generations at elevated temperature (25ºC), whereas the spermatogenesis-based effect of *mut-16* results in sterility after only one generation of heat stress (39). The germ cells of these sterile adult *mut-16(pk710)* mutant hermaphrodites exhibit abnormal P granule formation, indicating *Mutator*-dependent small RNAs play a key role in maintaining the integrity of RNAi pathways within the P granule, particularly at elevated temperature (39). However, the molecular and physiological defects that trigger the immediate onset of heat stress-induced sperm-based sterility in *mut-16* mutants remained unknown.

Here, we demonstrate that MUT-16, and thus *mutator* complex-dependent small RNA amplification, is required to properly coordinate and restrict the *mutator*-independent ALG-3/4 pathway function to the L4 developmental stage, particularly during stress. We leveraged comparative bioinformatic analyses on mRNA and small RNA changes in L4 and adult *mut-16* mutant and wild-type hermaphroditic animals cultured at permissive (20ºC) and elevated (25ºC) temperatures. We found sperm-enriched genes, which largely overlapped with ALG-3/4 pathway targets, are developmentally dysregulated in a small RNA-dependent manner in heat stressed *mut-16* mutant hermaphrodites. Further analyses revealed MUT-16 regulates expression of *alg-3* and *alg-4* across developmental stages during heat stress. Aberrant expression of the AGOs correlated with changes in 22G-RNA and 26G-RNA levels mapping to *alg-3* and *alg-4*. Additionally, we found the sperm-based sterility observed in heat stressed *mut-16* mutants is the consequence of severe spermiogenesis defects that phenocopy heat stressed *alg-3; alg-4* double mutants. Together, these results reveal an RNAi-to-RNAi regulatory cascade in which *mutator*-dependent RNAi pathways engage siRNA-mediated gene switches to coordinate *alg-3* and *alg-4* expression during the appropriate developmental timeframe. Thus, *mutator* complex-dependent small RNAs are essential for maintaining appropriate ALG-3/4 pathway function during stress, despite amplification of 22G-RNAs from ALG-3/4-targeted transcripts relying on EGO-1, and not the *mutator* complex.

## Materials and methods

### C. elegans strains

Unless otherwise stated, worms were grown at 20ºC on NGM plates seeded with OP50-1 *Escherichia coli* according to standard conditions (43). All strains are in the N2 background. Strains used include:

- N2 – wild-type.
- NL1810 – *mut-16(pk710) I*.
- BS553 – *fog-2(oz40) V*.
- DUP75 – *pgl-1(sam33[pgl-1::GFP::3xFLAG]) IV*.
- USC1252 – *mut-16(pk710) I; pgl-1(sam33[pgl-1::GFP::3xFLAG]) IV*.
- USC1092 – *alg-3(cmp153[GFP + loxP + 3xFLAG::alg-3]) IV*.
- RAK138 – *mut-16(pk710) I; alg-3(cmp153[GFP + loxP + 3xFLAG::alg-3]) IV*.
- JMC207 – *alg-4(tor143[GFP::3xFLAG::alg-4]) III*.
- RAK139 – *mut-16(pk710) I; alg-4(tor143[GFP::3xFLAG::alg-4]) III*.

### Male induction

L4s were plated on NGM plates and incubated at 30ºC for 4, 5 and 6 h then placed at 20ºC to recover. In the broods, males were isolated and mated with young adult hermaphrodites to maintain increased incidences of males within the populations.

### Mating brood size assay

Synchronized L1s of *fog-2(oz40)*, *pgl-1::GFP::3xFLAG*, *mut-16(pk710)*, and *mut-16(pk710); pgl-1::GFP::3xFLAG* male-containing populations were plated on NGM plates and cultured at 20ºC or 25ºC for a single generation. Mating plates were set up using individual L4 hermaphrodites of *fog-2(oz40)* or *mut-16(pk710)* grown at 20ºC or 25ºC and individual males of *pgl-1::GFP::3xFLAG* or *mut-16(pk710); pgl-1::GFP::3xFLAG* grown at 20ºC or 25ºC (ten plates per cross per temperature). Expression of PGL-1::GFP::3xFLAG was used to distinguish cross-progeny from the hermaphrodite's self-progeny. The number of GFP-expressing eggs laid were counted for each hermaphrodite.

### Fluorescence microscopy

Synchronized L1s were plated on NGM plates and cultured at 20°C and 25°C. Males were isolated as L4s and allowed to develop to adulthood for 24 h in the absence of hermaphrodites (to prevent mating). Whole animals were fixed in pre-chilled (–20ºC) methanol for 5 min then washed twice in 1xPBST before being incubated in 0.17 mg/ml DAPI solution in PBST for 15 min, followed by three washes in 1xPBST. Imaging was performed on an AxioImager.M2 (Zeiss) using a Plan-Apochromat 63×/1.4-oil immersion objective. For sperm counting, z-stacks were processed using FIJI (44,45) and a projection of the stack with sperm in different planes were pseudocolored using the hyperstacks temporal-color code function. The multipoint tool in FIJI was then used to count the post-meiotic sperm in each male. The stack projection was pseudo-colored using Adobe Photoshop. Post-meiotic sperm were counted for 5 biological replicates per genotype per condition. For GFP::3xFLAG::ALG-3 and GFP::3xFLAG::ALG-4 images, Z-stacks were acquired and pseudo-colored using Adobe Photoshop.

### Spicule morphology assay

Synchronized L1s of wild-type and *mut-16(pk710)* worms were plated on NGM plates and cultured at 20°C and 25°C. Males were collected as adults (∼68 h at 20°C and ∼48 h at 25°C) and were live imaged in M9 buffer. Imaging was performed on an AxioImager.M2 (Zeiss) using a Plan-Apochromat 63×/1.4-oil immersion objective. One spicule per tail was scored for spicule morphology (non-crumpled or crumpled) and spicule length. Spicule length was measured in FIJI using the line and measure tools (44,45). Between 40 and 51 spicules were assayed per genotype per temperature condition.

### RNA extraction

Synchronized L1s of wild-type and *mut-16(pk710)* worms were plated on enriched peptone plates and cultured at 20°C and 25°C. 8000 animals per sample were harvested as L4s (∼56 h at 20°C and ∼39 h at 25°C) for RNA extraction. Worms were washed off plates using water and then settled on ice to form a pellet. Water was aspirated off and worm pellets were resuspended in 1ml TRIzol reagent (Life Technologies) and freeze-thawed on dry ice followed by vortexing. Worm carcasses were pelleted using centrifugation and the supernatant containing RNA was collected. 0.2 volume chloroform was added to supernatant, vortexed, centrifuged, and then the aqueous phase was transferred to a new tube. Samples were precipitated using isopropanol and rehydrated in 50 μl nuclease-free H_2_O.

### mRNA-seq library preparation

Nuclease-free H_2_O was added to 7.5 μg of each total RNA sample, extracted from whole animals to a final volume of 100 μl. Samples were incubated at 65°C for 2 min then incubated on ice. The Dynabeads mRNA Purification Kit (ThermoFisher 61006) was used according to the manufacturer's protocol. 20μl of Dynabeads was used for each sample. 100ng of each mRNA sample was used to prepare libraries with the NEBNext Ultra II Directional RNA Library Prep Kit for Illumina (NEB E7760S) according to the manual, using NEBNext multiplex oligos for Illumina (NEB E7335S). Library quality was assessed (Agilent BioAnalyzer Chip) and concentration was determined using the Qubit 1× dsDNA HS Assay kit (ThermoFisher Q33231). Libraries were sequenced on the Illumina NextSeq500 (SE 75-bp reads) platform. Two biological replicates were generated for wild-type (N2) and *mut-16(pk710)* mutants cultured at 20°C and 25°C.

### Small RNA library preparation

Small RNAs (18- to 30-nt) were size selected on denaturing 15% polyacrylamide gels (Bio-Rad 3450091) from total RNA samples. Small RNAs were treated with 5′ RNA polyphosphatase (Epicenter RP8092H) and ligated to 3′ pre-adenylated adapter with Truncated T4 RNA ligase (NEB M0373L). Small RNAs were then hybridized to the reverse transcription primer, ligated to the 5′ adapter with T4 RNA ligase (NEB M0204L), and reverse transcribed with Superscript III (Thermo Fisher 18080–051) before being amplified using Q5 High-Fidelity DNA polymerase (NEB M0491L) and size selected on a 10% polyacrylamide gel (Bio-Rad 3450051). Library quality was assessed (Agilent BioAnalyzer Chip) and concentration was determined using the Qubit 1× dsDNA HS Assay kit (ThermoFisher Q33231). Libraries were sequenced on the Illumina NextSeq500 (SE 75-bp reads) platform. Two biological replicates were generated for wild-type (N2) and *mut-16(pk710)* mutants cultured at 20°C and 25°C.

### cDNA preparation and qRT-PCR

RNA samples were DNAse treated using DNAse I, Amplification Grade (Invitrogen 18068015), and reverse transcribed with SuperScript IV Reverse Transcriptase (Invitrogen 18090050), following manufacturers’ protocols. All Real time PCR reactions were performed using the PowerTrack SYBR Green Master Mix (Applied Biosystems A46109) and run on the QuantStudio 3 Real-Time PCR System (Applied Biosystems A28567). Samples were run with three technical replicates and three biological replicates and normalized to *rpl-32*. Primers used are listed in Supplemental Table S2. qRT-PCR Ct values are provided in the Source Data.

### *In vitro* sperm activation assay

Synchronized L1s of wild-type and *mut-16(pk710)* worms were plated on NGM plates and cultured at 20°C and 25°C. L4 males of wild-type and *mut-16(pk710)* worms were isolated on NGM plates without hermaphrodites and allowed to develop into adults at 20ºC and 25ºC for 24 h. 10–15 males were dissected in 15 μl sperm buffer (50mM HEPES, 50 mM NaCl, 25mM KCl, 5 mM CaCl_2_, 1 mM MgSO_4_, 0.1% BSA) with or without 400 ng/ml Pronase E (Millipore Sigma P8811) as described previously (46). Tails were nicked off to release the sperm, and the slides were incubated in a hybridization chamber for 15 min before a coverslip was mounted. Sperm were imaged on an AxioImager.M2 (Zeiss) using a Plan-Apochromat 63×/1.4-oil immersion objective. Pseudopod formation was scored for 200 sperm per genotype per temperature condition. Source Data is provided for pseudopod formation.

### Western blots

Synchronized L1s *C. elegans* were plated on NGM plates and cultured at 20°C and 25°C. For sample collection, animals were harvested as L4s (∼56 h at 20°C and ∼39 h at 25°C) and young adults (∼65 h at 20ºC and ∼47 h at 25ºC). Worms were washed off plates using water and then centrifuged. Worm pellets were resuspended in 2× SB buffer (20% glycerol, 12 mM Tris pH 6.8, 4% SDS, 1% bromophenol blue, 5% BME) and incubated at 95°C for 10 min, vortexing three times. Equal volume of each sample was loaded into 4–12% Bis–Tris polyacrylamide gels (Invitrogen NW04122BOX), transferred to nitrocellulose membranes (Invitrogen LC20001), blocked in 5% milk in PBST, and probed with anti-FLAG 1:1000 [M2 clone] (Sigma-Aldrich F1804) and anti-Tubulin 1:500 (Sigma-Aldrich T9026). Secondary HRP-conjugated anti-mouse IgG 1:10 000 (Abcam A16078) was used. To visualize, the membrane was treated with Super Signal West Pico PLUS Chemiluminescent Substrate (Thermo Scientific 34580) and imaged with a ChemiDoc MP Imaging System (Biorad). Quantification for relative density of band on western blots were performed using Image Lab Software (Biorad). Source Data is provided for western blots.

### Bioinformatic analysis of mRNA-seq and small RNA-seq libraries

For small RNA libraries, sequences were parsed from adapters using Cutadapt v.4.1(47) (options: -a TGGAATTCTCGGGTGCCAAGG -m 17 –nextseq-trim = 20 –max-n 2) and mapped to the *C. elegans* genome, WS258, using HISAT2 v.2.2.1(48) (options: -q -k 11 -t -p 8) and the transcriptome using Salmonv.1.9.0 (47) (default options). Differential expression analysis was performed using DESeq2 v.1.38.0 (48). Reads per million were plotted along the WS258 genome using Integrative Genomics Viewer 2.12.3 (49).

For mRNA libraries, sequences were parsed from adapters using Cutadapt v.4.1 (50) (options: -a AGATCGGAAGAGCACACGTCTGAACTCCAGTCA -m 17 –nextseq-trim = 20 –max-n 2) and mapped to the *C. elegans* genome, WS258, using HISAT2 v.2.2.1 (51) (options: -q -k 11 -t -p 8) and the transcriptome using Salmonv.1.9.0 (47) (default options). Differential expression analysis was performed using DESeq2 v.1.38.0 (48). A log_2_(fold change) >1 or less than –1 and an adjusted *P*-value ≤0.05 was used as a cutoff to identify up- and down-regulated genes in the DESeq2 output. Reads per million were plotted along the WS258 genome using Integrative Genomics Viewer 2.12.3 (49). Tissue enrichment analysis was performed using reference gene lists from WormExp (52,53).

Enrichment analyses used gene lists for RNAi pathways known to target sperm genes (CSR-1 target genes, ALG-3/4 target genes, ERGO-1 target genes and PRG-1 target genes), previously identified *mutator* target genes, or germline enriched genes (spermatogenesis-enriched genes, oogenesis-enriched genes, sex-neutral genes), and soma-enriched genes previously shown to be aberrantly expressed in heat-stressed *mut-16* mutant adults (muscle-enriched genes and neuron-enriched genes) (29,31,32,39,54–60). Additional data analysis was done using R, Excel and Python. Sequencing data is summarized in Supplemental Table S1. Principal Component Analysis and hierarchical clustering of global correlation coefficients between samples heat map analyses for mRNA and small RNA sequencing libraries used were performed with deepTools (61) and are provided in Supplemental Figures S6 and S7.

### Statistical analysis of qPCR reactions

For each qPCR experiment, *n* = 3 biological replicates, with three technical replicates, for each condition were examined. Statistical parameters, including log_2_(fold change), normalized as indicated, standard deviation, and statistical significance are reported in the figures.

## Results

### Differentially expressed genes in heat stressed *mut-16* mutant L4s are enriched for sperm genes

To further understand the temperature-sensitive loss of sperm-based fertility in *mut-16* mutants, we sought to identify the mRNA expression changes that occur in heat-stressed hermaphrodites during the L4 developmental stage, when spermatogenesis should occur. To this end, we extracted total RNA from synchronized L4 wild-type and *mut-16* mutant hermaphrodites cultured at 20ºC and 25ºC for a single generation and generated mRNA-seq libraries and size selected small RNA-seq libraries. We used differential expression analyses to identify genes whose mRNA levels changed due to elevated temperature (25ºC), the *mut-16* mutation, and the combination of both. Then, we established a list of genes that were significantly up- or down-regulated (425 and 81 genes, respectively) exclusively in *mut-16* mutants cultured at 25ºC, omitting any genes with differential expression due to heat stress or the *mut-16* mutation alone.

**Figure 1.**
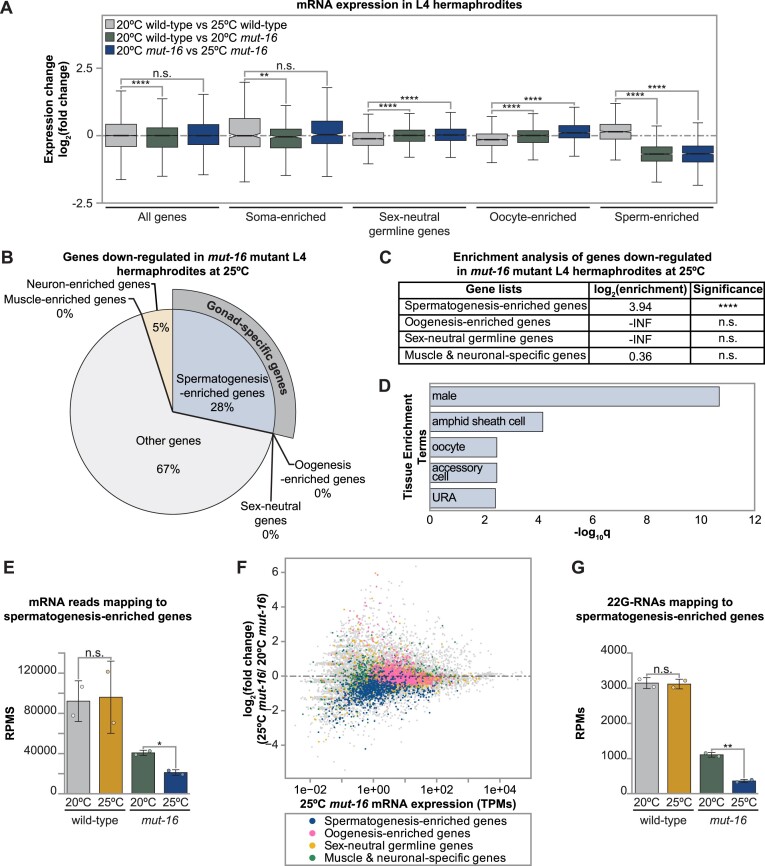
Differentially expressed genes in heat stressed *mut-16* mutant L4s are enriched for sperm genes. (**A**) Box plots depicting mRNA expression change for published enriched gene sets, in log_2_(fold change), for comparisons between wild-type L4 hermaphrodites cultured at 20ºC and 25ºC (gray), wild-type and *mut-16* mutant L4 hermaphrodites cultured at 20ºC (green), and *mut-16* mutant L4 hermaphrodites cultured at 20ºC and 25ºC (blue). Notches indicate the 95% confidence interval of the median, bolded midline indicates median value, box indicates the first and third quartiles, and whiskers represented the most extreme data points within 1.5 times the interquartile range, excluding outliers. Wilcoxon tests were performed to determine statistical significance between the log_2_(fold change) upon heat stress in wild-type L4s compared to the log_2_(fold change) due to the *mut-16* mutation and due to the *mut-16* mutation at 25°C, and *p* values were adjusted for multiple comparisons. (**B**) Percentage of gonad-specific and non-gonad-specific genes represented in the genes down-regulated exclusively in *mut-16* mutant L4s at 25°C. (**C**) Enrichment analysis for spermatogenesis, oogenesis, sex-neutral genes, and muscle-specific and neuronal-specific genes amongst the genes down-regulated during heat stress in *mut-16* mutants. Two-tailed *p* values for enrichment or depletion were calculated using the Fisher's exact test function in R. -INF indicates there were no down-regulated genes overlapping with the gene list. (**D**) Tissue enrichment analysis was performed using WormExp (log_10_Q ≥ 2 and FDR < 0.05) for genes down-regulated exclusively in *mut-16* mutants at 25°C. (**E**) mRNA transcripts mapping to spermatogenesis-enriched genes are counted, in reads per million (RPMs), for wild-type and *mut-16* mutant L4 animals cultured at 20°C and 25°C. (**F**) Shown is difference in expression (log_2_(fold change)) for genes in *mut-16* mutants at 25ºC compared to *mut-16* mutants at 20ºC plotted according to the average abundance of normalized reads (in transcripts per million (TPMs)) in libraries from *mut-16* mutant L4 hermaphrodites grown at 25ºC. Each dot represents a gene, with sperm-enriched genes highlighted in blue, oogenesis-enriched genes highlighted in pink, sex-neutral germline genes highlighted in yellow, and muscle and neuronal genes highlighted in green. (**G**) 22G-RNAs mapping to spermatogenesis-enriched genes are counted, in reads per million (RPMs), for wild-type and *mut-16* mutant L4 hermaphrodites cultured at 20°C and 25°C. (A–G) For each genotype and condition, two biological replicates were sequenced. (E, G) Bar graphs represent the mean with dots representing summed RPMs for biological replicates and error bars indicating standard deviation. Two-tailed Welch's *t*-tests were performed to determine statistical significance. (A, C, E, G) n.s. denotes not significant and indicates a *P*-value > 0.05, * indicates a *P*-value ≤ 0.05, ** indicates a *P*-value ≤ 0.01, *** indicates a *P*-value ≤ 0.001, and **** indicates a *P*-value ≤ 0.0001.

Our prior work in adult *mut-16* hermaphrodites indicated that heat stress induces aberrant expression of sperm-enriched and somatic genes, and reduced expression of oogenesis-enriched and sex-neutral germline genes, within the germline (39). To determine if similar changes in gene expression correlated with heat stress in L4 and adult *mut-16* mutants, we assessed the enrichment of germline-specific (sperm-enriched, oogenesis-enriched, and sex-neutral) genes and somatic (muscle and neuronal) genes. In contrast to our previous findings, the 425 genes up-regulated exclusively in *mut-16* mutant L4 hermaphrodites cultured at 25ºC were slightly enriched for oogenesis and sex-neutral germline genes but not somatic (muscle and neuronal) or spermatogenesis genes (Figure 1A, Supplementary Figure S1A and B). We confirmed somatic (muscle and neuronal) genes are not up-regulated in L4 *mut-16* mutants using qRT-PCR (Supplementary Figure S1C). This suggests aberrant expression of somatic genes within the germline is specific to the loss of germ cell fate in adult heat-stressed *mut-16* mutants.

Surprisingly, our analyses indicated sperm genes were enriched amongst the genes down-regulated exclusively in heat stressed *mut-16* mutant L4 hermaphrodites, who should be undergoing spermatogenesis (Figure 1B and C). Tissue enrichment analysis indicated these down-regulated genes are typically expressed in the male tissues of *C. elegans* (Figure 1D). Next, we assessed the mRNA levels of all spermatogenesis-enriched genes in L4 hermaphrodites. We found that heat stress alone does not significantly impact the expression of spermatogenesis-enriched genes in wild-type L4 hermaphrodites (Figure 1E). However, sperm gene transcript levels are significantly down-regulated in L4 *mut-16* mutant hermaphrodites compared to wild-type animals at 20ºC (Figure 1A and E). The reduction of sperm gene expression in L4 hermaphroditic *mut-16* mutants was further exacerbated by heat stress (Figure 1A, E and F). We corroborated this using qRT-PCR (Supplementary Figure S1D). The finding that sperm gene expression is reduced in *mut-16* mutant L4s contrasts with our previous observation that the *mut-16* mutation leads to up-regulation of sperm genes in the germline of adult animals (39) and indicates the effect of *mut-16* on sperm gene regulation depends on the animal's developmental stage.

Our previous work found that increased expression of spermatogenesis genes in hermaphrodite adults is independently triggered by heat stress and loss of MUT-16-dependent 22G-RNAs (39). To determine whether the altered expression of spermatogenesis genes in *mut-16* L4 hermaphrodites occurred in a small RNA-dependent manner, we assessed 22G-RNA levels mapping to all sperm genes. We found that elevated temperature does not alter 22G-RNA levels mapping to sperm genes in wild-type L4 hermaphrodites (Figure 1G). However, disruption of the *mutator* complex in *mut-16* mutants does trigger a reduction in 22G-RNA-targeting of sperm gene transcripts, which was further exacerbated by heat stress (Figure 1G). These data indicate that during the L4 developmental stage, the expression of spermatogenesis genes is susceptible to perturbations in MUT-16-dependent 22G-RNA small RNA populations. Furthermore, growth at elevated temperature exacerbates the effect of the *mut-16* mutation, suggesting that MUT-16-dependent small RNA amplification plays a role in mitigating the effects of heat stress on sperm gene expression. Moving forward in our study of the temperature-sensitive sperm-based fertility, we focused on the genes down-regulated exclusively in *mut-16* mutant L4 hermaphrodites grown at elevated temperature (25ºC).

### ALG-3/4 pathway targets are enriched amongst genes down-regulated in heat stressed *mut-16* mutant L4s

**Figure 2.**
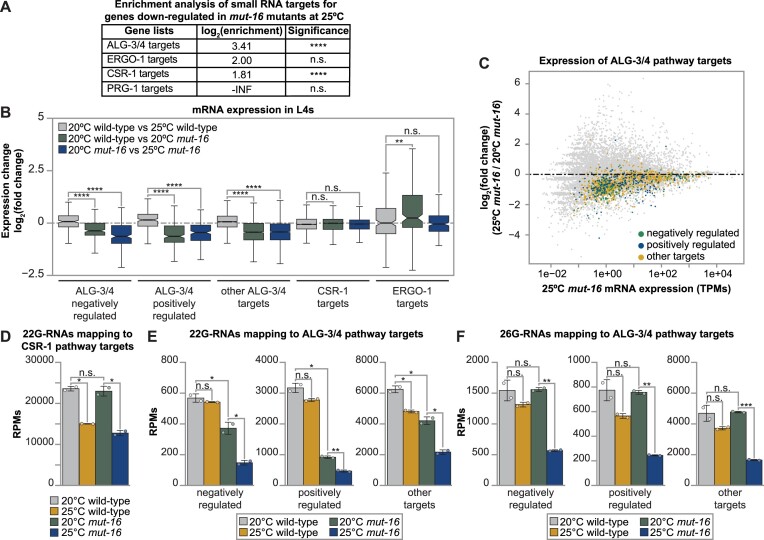
ALG-3/4 pathway targets are enriched amongst genes down-regulated in heat stressed *mut-16* mutant L4s. (**A**) Enrichment analysis for ALG-3/4, ERGO-1, CSR-1 and piRNA pathway targets amongst the genes down-regulated during heat stress in *mut-16* mutants is shown. Two-tailed *p* values for enrichment or depletion were calculated using the Fisher's exact test function in R. -INF indicates there were no down-regulated genes overlapping with the gene list. (**B**) Comparison of expression changes in wild-type L4s cultured at 20ºC compared to wild-type L4s cultured at 25ºC (gray), wild-type L4s cultured at 20ºC compared to *mut-16* mutant L4s cultured at 20ºC (green), and *mut-16* mutant L4s cultured at 20ºC compared to *mut-16* mutant L4s cultured at 25ºC (blue) for published ALG-3/4 pathway and CSR-1 pathway target gene lists. Notches indicate the 95% confidence interval of the median, bolded midline indicates median value, box indicates the first and third quartiles, and whiskers represented the most extreme data points within 1.5 times the interquartile range, excluding outliers. Wilcoxon tests were performed to determine statistical significance between the log_2_(fold change) upon heat stress in wild-type L4s compared to the log_2_(fold change) due to the *mut-16* mutation and due to the *mut-16* mutation at 25°C, and *P* values were adjusted for multiple comparisons. (**C**) Shown is difference in expression (log_2_(fold change)) for genes in *mut-16* mutants at 25ºC compared to *mut-16* mutants at 20ºC plotted according to the average abundance of normalized reads (in transcripts per million (TPMs)) in libraries from *mut-16* mutant L4 hermaphrodites grown at 25ºC. Each dot represents a gene, with negatively regulated ALG-3/4 pathway targets (green), positively regulated ALG-3/4 pathway targets (blue), and other ALG-3/4 pathway targets (gold) highlighted. (**D**) 22G-RNAs mapping to CSR-1 pathway target genes, (**E**) 22G-RNAs mapping to negatively regulated, positively regulated, and other ALG-3/4 pathway target genes, and (**F**) 26G-RNAs mapping to negatively regulated, positively regulated, and other ALG-3/4 pathway target genes are counted, in reads per million (RPMs), for wild-type and *mut-16* mutant L4 hermaphrodites cultured at 20°C and 25°C. (A–F) For each genotype and condition, two biological replicates were sequenced. (D–F) Bar graphs represent the mean with dots representing summed RPMs for biological replicates and error bars indicating standard deviation. Two-tailed Welch's *t*-tests were performed to determine statistical significance. n.s. denotes not significant and indicates a *P*-value > 0.05, * indicates a *P*-value ≤ 0.05, ** indicates a *P*-value ≤ 0.01, *** indicates a *P*-value ≤ 0.001 and **** indicates a *P*-value ≤ 0.0001.

As several RNAi pathway branches (ALG-3/4, ERGO-1, CSR-1 and PRG-1) are known to target sperm genes, we assessed the enrichment of different branches’ targets amongst the 81 genes down-regulated exclusively in *mut-16* mutant L4 hermaphrodites at 25ºC. Surprisingly, the down-regulated genes were not enriched for targets of endogenous RNAi pathways that rely on the *mutator* complex for 22G-RNA amplification (ERGO-1 and PRG-1), but rather for targets of the *mutator* complex-independent ALG-3/4 and CSR-1 pathways (Figure 2A). The previously established ALG-3/4 pathway targets and CSR-1 pathway targets overlap considerably (798 CSR-1 and ALG-3/4 regulated targets, Supplementary Figure S2A), so we looked more closely at the CSR-1 pathway targets present in the list of genes down-regulated specifically in *mut-16* mutants at 25ºC. We found that most of them (17 out of 22 genes) are also annotated as ALG-3/4 pathway targets, indicating targets unique to the CSR-1 pathway are not enriched amongst the genes down-regulated exclusively in *mut-16* mutant L4 hermaphrodites at 25ºC. 26 of the 81 genes down- regulated exclusively in *mut-16* mutant L4 hermaphrodites at 25ºC are annotated as ALG-3/4 pathway targets. There is a small subset of *C. elegans* genes that are annotated as targets of both *mutator*-dependent 22G-RNAs (ERGO-1-class, WAGO-class, and PRG-1-class) and ALG-3/4 (Supplementary Figure S2A); however, these genes are not amongst the list of genes down-regulated specifically in heat stressed *mut-16* mutant L4 hermaphrodites.

As the *mutator* complex does not canonically function in the ALG-3/4 pathway, or the downstream CSR-1 pathway, we next characterized the impact of heat stress, loss of the *mutator* complex, and the combination of both on these pathways’ targets. ALG-3/4 pathway targets can be classified by the effect of ALG-3/4-targeting on their expression: some are negatively regulated by ALG-3/4 while others are positively regulated, and some targets can be immunoprecipitated with ALG-3/4 but do not have altered expression in *alg-3/4* mutants (32). We assessed the mRNA levels of all genes categorized as negatively regulated ALG-3/4 targets, positively regulated ALG-3/4 targets, ‘other’ ALG-3/4 pathway targets, CSR-1 pathway targets, and ERGO-1 pathway targets (204, 214, 988, 3599 and 288 genes, respectively) in wild-type and *mut-16* mutant L4 hermaphrodites. We found the *mut-16* mutation results in overall reduced expression for the three groups of ALG-3/4 targets (Figure 2B). Elevated temperature (25ºC) further exacerbates the effect of the *mut-16* mutation on all three ALG-3/4 pathway target groups in L4 hermaphrodites (Figure 2B and C). ALG-3/4 pathway targets were also significantly depleted from the list of genes exclusively up-regulated in *mut-16* mutant L4 hermaphrodites at 25ºC, further corroborating the negative effect of the *mut-16* mutation on their expression during heat stress (Supplementary Figure S2B and C). Overall expression of CSR-1 pathway targets was not significantly affected by heat stress, the *mut-16* mutation, or the combination of both (Figure 2B). This is not surprising because despite 27% of the genes exclusively down-regulated in heat-stressed *mut-16* mutant L4 hermaphrodites being annotated as CSR-1 targets or overlapping CSR-1 and ALG-3/4 targets, those genes represent only 0.6% of all CSR-1 targets (Supplementary Figure S2A). Further, ERGO-1 pathway targets, which were not significantly enriched amongst the down-regulated genes, showed no significant change in mRNA levels in the heat stressed *mut-16* mutants (Figure 2B). Together, these data suggest the ALG-3/4 pathway is the major contributor to the small RNA-mediated effect on sperm genes in *mut-16* mutant L4 animals raised at 25ºC.

Next, we assessed changes in small RNAs mapping to targets of the *mutator* complex-independent ALG-3/4 and CSR-1 pathways. As expected, 22G-RNA levels mapping to the CSR-1 pathway targets were not impacted by loss of *mut-16* (Figure 2D). However, heat stress triggered loss of 22G-RNAs mapping to CSR-1 targets in wild-type and *mut-16* mutant animals, suggesting CSR-1-loaded 22G-RNAs are susceptible to elevated temperature (Figure 2D). In contrast, 22G-RNAs mapping to ALG-3/4 targets were not significantly affected by heat stress in wild-type L4 animals (Figure 2E). Yet, the *mut-16* mutation caused moderate to severe reduction in 22G-RNAs mapping to the three ALG-3/4 target groups (Figure 2E). The combination of heat stress and loss of *mutator* complex-dependent 22G-RNA amplification had an additive effect, resulting in severely reduced numbers of 22G-RNAs mapping to ALG-3/4 targets in heat-stressed *mut-16* mutant L4 hermaphrodites (Figure 2E). As ALG-3/4 bind 26G-RNAs, we examined the levels of the 26G-RNAs mapping to the three ALG-3/4 target groups. We found that neither heat stress nor the *mut-16* mutation alone led to a significant loss of 26G-RNAs mapping to the three groups of ALG-3/4 targets (Figure 2F). However, the combination of heat stress and the *mut-16* mutation did trigger a severe loss of 26G-RNAs mapping to all three ALG-3/4 target groups (Figure 2F). Our analyses indicate that 22G-RNAs targeting CSR-1 and ALG-3/4 pathway targets are susceptible to heat stress. While it has been previously observed that 22G-RNAs mapping to some ALG-3/4 targets are depleted in *mut-16* mutant adult hermaphrodites at 20ºC (31), this is the first evidence indicating the *mutator* complex is critical for maintaining 22G- and 26G-RNA levels mapping to ALG-3/4 targets during the L4 stage, when the ALG-3/4 pathway functions. Together, our differential expression analyses indicate that MUT-16 plays a role in maintaining homeostatic levels of 22G-RNAs and 26G-RNAs mapping to ALG-3/4 pathway targets and appropriate L4-stage expression of ALG-3/4 targets, particularly during heat stress.

### *Mutator* complex-dependent small RNAs regulate *alg-3* and *alg-4* expression during heat stress

**Figure 3.**
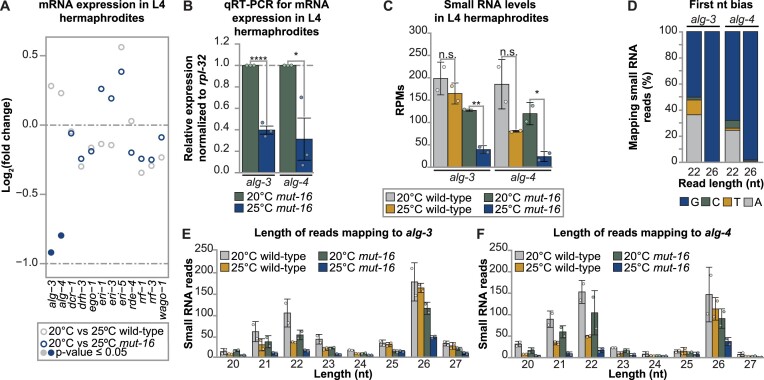
*Mutator* complex-dependent small RNAs are required for *alg-3* and *alg-4* expression during heat stress in L4s. (**A**) Strip plot showing the change in expression of ALG-3/4 pathway factors. Circles represent the log_2_(fold change) for each gene, as determined by DESeq2, for comparisons between wild-type L4 hermaphrodites cultured at 20ºC and 25ºC (gray) and *mut-16* mutant L4 hermaphrodites cultured at 20ºC and 25ºC (blue). Filled circles indicate a significant *p* value as determined by DESeq2 (*P*-value ≤ 0.05). (**B**) qRT-PCR assay of *alg-3* and *alg-4* expression, normalized to *rpl-32* expression, in *mut-16* mutant L4 hermaphrodites cultured at 25ºC normalized to expression levels in *mut-16* mutant L4 hermaphrodites cultured at 20ºC. *n* = 3 biological replicates. (**C**) Total small RNA reads per million (RPMs) mapping to *alg-3* and *alg-4* in wild-type and *mut-16* mutant L4 hermaphrodites cultured at 20°C and 25°C. (**D**) Shown is the percentage of 22-nt and 26-nt reads mapping to *alg-3* and *alg-4* with A, T, C or G represented in the first position of the read in wild-type L4 animals cultured at 20ºC. (**E**) Shown are size profiles of all reads mapping to the *alg-3* genomic locus in wild-type and *mut-16* mutant L4 animals cultured at 20°C and 25°C. (**F**) Shown are size profiles of all reads mapping to the *alg-4* genomic locus in wild-type and *mut-16* mutant L4 animals cultured at 20°C and 25°C. (A–F) For each genotype and condition, two biological replicates were sequenced. (B, C, E, F) Bar graphs represent the mean with dots representing each biological replicate and error bars indicating standard deviation. For (B, C) two-tailed student *t*-tests were performed to determine statistical significance. n.s. denotes not significant and indicates a *P*-value > 0.05, * indicates a *P*-value ≤ 0.05 and ** indicates a *P*-value ≤ 0.01.

It should be noted that 22G-RNAs are impacted in *mut-16* mutants at 20ºC and 25ºC, whereas 26G-RNAs mapping to ALG-3/4 pathway targets are specifically affected in the heat-stressed *mut-16* mutants. One possible explanation is the effect on 26G-RNAs is a consequence of reduced 22G-RNAs in the heat-stressed *mut-16* mutants. Previously, it was proposed that initiation of ALG-3/4 26G-RNA production relies on the presence of CSR-1-loaded 22G-RNAs inherited from the previous generation (32). Thus, disrupting 22G-RNA levels would impact the feed-forward mechanism required to amplify ALG-3/4-class 26G-RNAs. Another possibility that we explore here is reduced expression of the AGOs that load the 26G-RNAs driving decreased production and/or stability of the 26G-RNAs in heat-stressed *mut-16* mutants. To this end, we assessed the expression of known ALG-3/4 pathway factors. We found the two primary Argonaute proteins, ALG-3 and ALG-4, had significantly reduced expression in *mut-16* mutant L4 hermaphrodites experiencing heat stress (Figure 3A, B, Supplementary Figure S3A, and B). In contrast, in heat-stressed *mut-16* mutants we did not see significant changes in the mRNA levels of the AGOs *ergo-1* and *csr-1*, which also regulate sperm gene transcripts (Supplementary Figure S3C, D, and E). These data indicate that the transcript levels of *alg-3/4*, and not of other AGOs regulating sperm genes, are down-regulated in heat stressed *mut-16* mutants during the L4 stage. We next sought to determine if changes in *alg-3* and *alg-4* expression occurred in a small RNA-dependent manner. To this end, we examined the small RNA reads mapping to *alg-3* and *alg-4*. In wild-type L4 hermaphrodites, small RNAs target both *alg-3* and *alg-4* during the L4 developmental stage (Figure 3C, Supplementary Figure S3A, and B). Heat stress and the *mut-16* mutation each independently triggered slight reductions in small RNA levels mapping to *alg-3* and *alg-4*; however, the combination of both resulted in a severe loss of small RNAs targeting *alg-3* and *alg-4* (Figure 3C, Supplementary Figure S3A, and B). To further characterize these small RNAs, we examined their read lengths and first nucleotide (nt) biases. We found that *alg-3* and *alg-4* are predominately targeted by a group of 22G-RNAs and a group of 26G-RNAs (Figure 3D–F). It should be noted that 21-nt reads mapping to *alg-3* and *alg-4* largely have a 5′ G bias, suggesting these are 22G-RNAs that may have been shortened to 21-nt during reading trimming (Figure 3E, F, and Supplementary Figure S3F). In wild-type animals, elevated temperature (25ºC) resulted in a strong loss of 22G-RNAs, but not 26G-RNAs, targeting *alg-3* and *alg-4* (Figure 3E and F). The *mut-16* mutation alone triggered a minor loss of 22G-RNAs and 26G-RNAs targeting *alg-3* and *alg-4*; however, the combination of heat stress and the *mut-16* mutation resulted in severe depletion of both 22G-RNAs and 26G-RNAs mapping to *alg-3* and *alg-4* (Figure 3E and F). Our data indicates that L4 stage expression of *alg-3* and *alg-4* is regulated in a small RNA-dependent manner. Moreover, our data suggests that the *mutator* complex is critical for maintaining small RNA-mediated regulation of *alg-3* and *alg-4* during stressful conditions.

### *alg-3* and *alg-4* expression is developmentally dysregulated in heat stressed *mut-16* mutants

**Figure 4.**
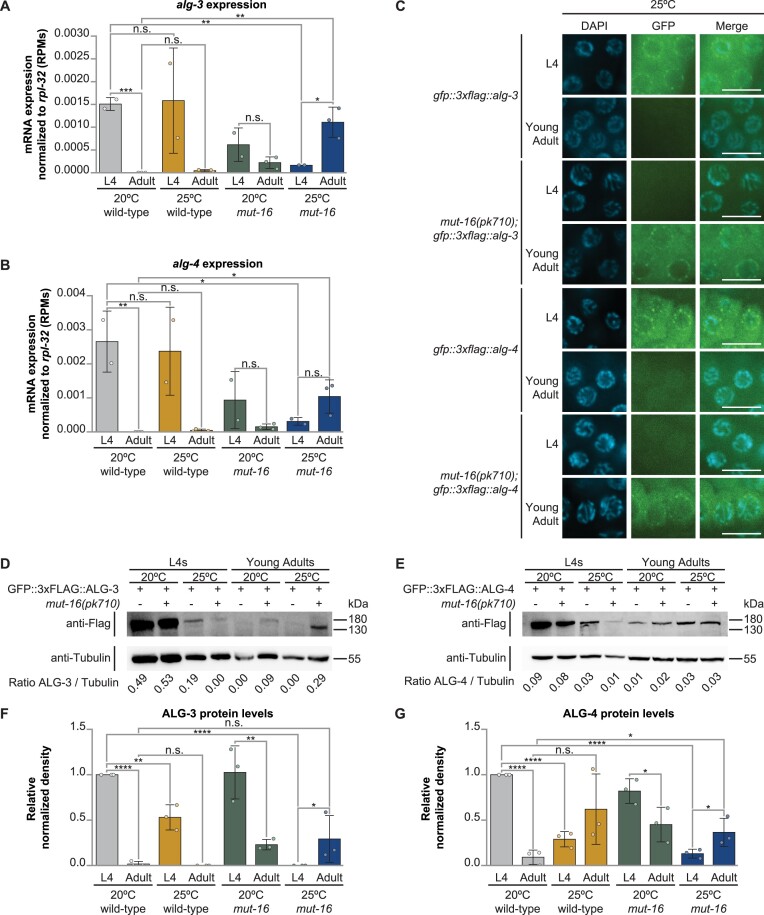
*alg-3* and *alg-4* expression is developmentally dysregulated in heat stressed *mut-16* mutants. Bar graphs depicting mRNA transcripts mapping to (**A**) *alg-3* and (**B**) *alg-4*, normalized to the expression level of *rpl-32*, in reads per million (RPMs), for L4 and adult wild-type and *mut-16* mutant hermaphrodites cultured at 20°C and 25°C.(A, B) Bar graphs represent the mean with dots representing summed RPMs for biological replicates and error bars indicating standard deviation. (**C**) Representative fluorescence microscopy images of germline nuclei from the proximal end of gonads of L4 and young adult stage animals expressing GFP::3XFLAG::ALG-3 or GFP::3XFLAG::ALG-4 in the wild-type and *mut-16* mutant background grown at 25ºC. Scale bars indicate 10 μm. Representative western blots for (**D**) GFP::3XFLAG::ALG-3 and (**E**) GFP::3XFLAG::ALG-4 in L4 and young adult stage animals expressing GFP::3XFLAG::ALG-3 or GFP::3XFLAG::ALG-4 in the wild-type and *mut-16* mutant background grown at 20ºC and 25ºC are shown. Also shown are bar graphs representing the quantification of biological replicates of the (**F**) GFP::3XFLAG::ALG-3 and (**G**) GFP::3XFLAG::ALG-4 western blots. (A, B) For each genotype and condition, two biological replicates were sequenced for L4-stage animals and three biological replicates were sequenced for adult-stage animals. (D–G) For each genotype, development stage, and condition, *n* = 3. (F, G) Bar graphs represent the mean with dots representing the relative density for biological replicates and error bars indicating standard deviation. For (A, B, F, G) one-tailed student *t*-tests were performed to determine statistical significance. n.s. denotes not significant and indicates a *P*-value > 0.05, * indicates a *P*-value ≤ 0.05, ** indicates a *P*-value ≤ 0.01, *** indicates a *P*-value ≤ 0.001, and **** indicates a *P*-value ≤ 0.0001.

As we observed reduced expression of *alg-3* and *alg-4* during the L4 stage, when the ALG-3/4 pathway should be functioning, we next sought to determine if *alg-3* and *alg-4* is developmentally dysregulated in heat stressed *mut-16* mutants. To this end, we used our previously published mRNA-seq and small RNA-seq libraries generated from adult wild-type and *mut-16* mutant hermaphrodites grown at 20ºC and 25ºC to plot the expression levels of *alg-3* and *alg-4* across the L4 and adult developmental stages. To account for potential differences when comparing L4 and adult mRNA samples, we normalized the expression levels to *rpl-32*, which encodes a ribosomal protein subunit and is expressed at comparable levels in wild-type and *mut-16* mutant hermaphrodites throughout the L4 and adult stages (Supplementary Figure S4A and B). We found *alg-3* and *alg-4* are down-regulated during the L4 stage and then aberrantly up-regulated during adulthood in heat stressed *mut-16* mutant hermaphrodites (Figures 4A, B and S4C). Increased targeting by *mutator* complex-independent 22G-RNAs correlated with the dysregulation of *alg-3* and *alg-4* in heat stressed *mut-16* mutant adults (Supplementary Figure S4C and D). However, while there were increased 26G-RNAs targeting *alg-3* and *alg-4* in *mut-16* mutants compared to wild-type adults at 20ºC, there was not a significant difference in 26G-RNAs targeting *alg-3* and *alg-4* in *mut-16* mutant adults at 20ºC compared to 25ºC (Supplementary Figure S4E). This finding suggests the disruption of 22G-RNA levels, and not 26G-RNA levels, may play a primary role in the dysregulation of *alg-3* and *alg-4* expression levels specifically during heat stress in *mut-16* mutants.

To move beyond correlation of trans-developmental sequencing data, we sought to determine if differences in functional protein levels for ALG-3 and ALG-4 could be observed in heat-stressed *mut-16* mutant L4 and adult hermaphrodites. To this end, we collected L4 and young adult hermaphrodites with ALG-3 or ALG-4 tagged with GFP::3xFLAG under the genes’ endogenous promoters in the wild-type or *mut-16* mutant background at 20ºC and 25ºC and completed germline imaging and protein sample collection. We performed western blotting using anti-FLAG for ALG-3 or ALG-4 and anti-Tubulin, as a control, and quantified the relative density of ALG-3, ALG-4 and Tubulin. As expected, we saw abundant protein levels of ALG-3 and ALG-4 in the L4 stage of wild-type animals grown at 20ºC and 25ºC, which localized to distinct foci at the periphery of the germ cell nuclei (Figure 4C–G, and Supplementary Figure S4F). Comparing samples from young adults to L4s, the protein levels of ALG-3 and ALG-4 were drastically reduced in wild-type animals grown at 20ºC and 25ºC and depleted from perinuclear germline foci; however, some ALG-4 protein was detected on the western blot in samples from young adult animals (Figure 4C–G, and Supplementary Figure S4F). Similarly, we saw that *mut-16* hermaphrodites grown at 20ºC had high levels of ALG-3 and ALG-4 protein that localized to perinuclear germ foci during the L4 stage and reduced protein levels in the young adult stage (Figure 4D–G, and Supplementary Figure S4F). However, *mut-16* hermaphrodites grown at 25ºC had little to no detectable ALG-3 and ALG-4 protein during the L4 stage and increased protein levels that localized to perinuclear germ foci in the young adult stage (Figure 4C–G). Together, these data corroborate the developmental dysregulation of ALG-3 and ALG-4 we observed at the mRNA level in heat-stressed *mut-16* mutants.

### Sperm-enriched genes are developmentally dysregulated in heat stressed *mut-16* mutants

**Figure 5.**
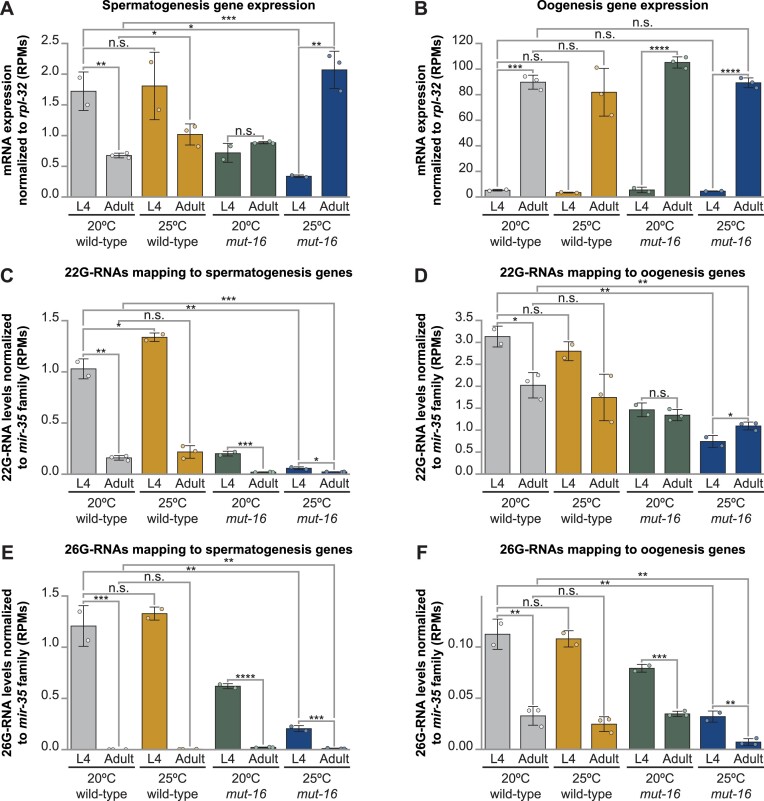
Sperm-enriched genes are developmentally dysregulated in heat stressed *mut-16* mutants. Bar graphs depicting mRNA transcripts mapping to (**A**) spermatogenesis-enriched genes and (**B**) oogenesis genes, normalized to the expression level of *rpl-32*, in reads per million (RPMs), for L4 and adult wild-type and *mut-16* mutant hermaphrodites cultured at 20°C and 25°C. Bar graphs depicting 22G-RNAs mapping to (**C**) spermatogenesis-enriched genes and (**D**) oogenesis-enriched genes, and 26G-RNAs mapping to (**E**) spermatogenesis-enriched genes and (**F**) oogenesis-enriched genes, normalized to all reads mapping to the *mir-35* family, in reads per million (RPMs), for L4 and adult wild-type and *mut-16* mutant hermaphrodites cultured at 20°C and 25°C. For (A–F) Bar graphs represent the mean with dots representing summed RPMs for biological replicates and error bars indicating standard deviation. For each genotype and condition, two biological replicates were sequenced for L4-stage animals and three biological replicates were sequenced for adult-stage animals. One-tailed student *t*-tests were performed to determine statistical significance. n.s. denotes not significant and indicates a *P*-value > 0.05, * indicates a *P*-value ≤ 0.05, ** indicates a *P*-value ≤ 0.01, *** indicates a *P*-value ≤ 0.001, and **** indicates a *P*-value ≤ 0.0001.

In the hermaphroditic germline of *C. elegans*, spermatogenesis and oogenesis occur during specific developmental time points within discrete regions of the same gonadal tissue. In wild-type animals, the ALG-3/4 pathway functions during the L4 stage to maintain proper transcript levels for spermatogenesis-enriched genes to ensure thermotolerant male fertility (25). As we saw developmental mis-coordination of *alg-3* and *alg-4* in heat stressed *mut-16* mutants, we wanted to determine whether dysregulation of spermatogenesis genes across developmental stages was also occurring. To this end, we examined expression profiles of sperm genes normalized to *rpl-32*, as described above. As expected, we found that, in wild-type animals, spermatogenesis genes are highly expressed during the L4 stage before being down-regulated in the adult animal when oogenesis genes are turned on (Figure 5A and B). Elevated temperature (25ºC) in wild-type animals did cause a slight increase in both spermatogenesis and oogenesis gene transcript levels during adulthood; however, the trend in expression across developmental stages was not altered (Figure 5A and B). Compared to wild-type hermaphrodites at 20ºC, *mut-16* mutants have reduced expression of sperm genes during the L4 stage and slightly elevated sperm gene expression during the adult stage (Figure 5A). Strikingly, heat stress exacerbated the effects of the *mut-16* mutation – causing a further reduction in sperm gene expression during the L4 stage and a dramatic increase in their expression during adulthood (Figure 5A). We did not observe a significant change in the expression levels of oogenesis genes across developmental stages in *mut-16* mutants at 20ºC or 25ºC (Figure 5B).

Next, we assessed 22G-RNA and 26G-RNA populations mapping to spermatogenesis-enriched and oogenesis-enriched genes across developmental stages to determine whether changes in the small RNA levels correlated with the observed changes in mRNAs. To control for levels of gonad tissue-derived RNA within total RNA samples due to developmental differences in germline size, we normalized the levels of 22G-RNAs and 26G-RNAs to all small RNA reads mapping to the germline-specific *mir-35* family (*mir-35–42*), which are not amplified in the *mutator* complex. Our analyses revealed that 22G-RNAs mapping to both spermatogenesis and oogenesis genes are strongly depleted throughout development in *mut-16* mutants and that this effect is exacerbated by heat stress (Figure 5C and D). This result is expected as *mut-16* mutants lack the ability to perform *mutator* complex-dependent 22G-RNA amplification; however, it is noteworthy that heat stress worsens the loss of 22G-RNAs. This suggests that the *mutator* complex plays an important role in mitigating the effects of heat stress on the pools of 22G-RNAs synthesized by MUT-16-independent mechanisms. Interestingly, 26G-RNAs mapping to both spermatogenesis and oogenesis genes in *mut-16* mutants were reduced during the L4 stage. Heat stress caused further loss of 26G-RNAs in *mut-16* mutant hermaphrodites mapping to both spermatogenesis and oogenesis genes during the L4 stage and a reduction in 26G-RNAs targeting oogenesis genes during adulthood (Figure 5E and F). Ultimately, our analyses indicate that the expression profile of oogenesis-enriched genes throughout development is not significantly impacted by elevated temperature (25ºC), changes in small RNA populations in *mut-16* mutants, or the combination of both. However, our analyses indicate that the effect of the *mut-16* mutation on spermatogenesis-enriched gene expression in hermaphrodites is dictated by the developmental stage of the animal. Based on our data, when *C. elegans* are grown at elevated temperature, *mutator* complex amplification of 22G-RNAs is required for proper expression of sperm genes in germ cells fated to be sperm during the L4 stage. Then during adulthood, *mutator* complex amplification of 22G-RNAs is required for silencing of sperm genes in germ cells fated to be oocytes. This is the first evidence that the *mutator* complex plays a role in mitigating the effects of heat stress on the levels of 26G-RNAs mapping to sperm genes and proper sperm gene expression during the L4 stage in hermaphrodites. Moreover, our data suggests that despite the spermatogenesis-to-oogenesis switch occurring within the same gonadal tissue, these developmental programs are independently regulated at the transcript level.

### Spermiogenesis defects trigger the onset of temperature-sensitive sperm-based sterility in *mut-16* mutants

**Figure 6.**
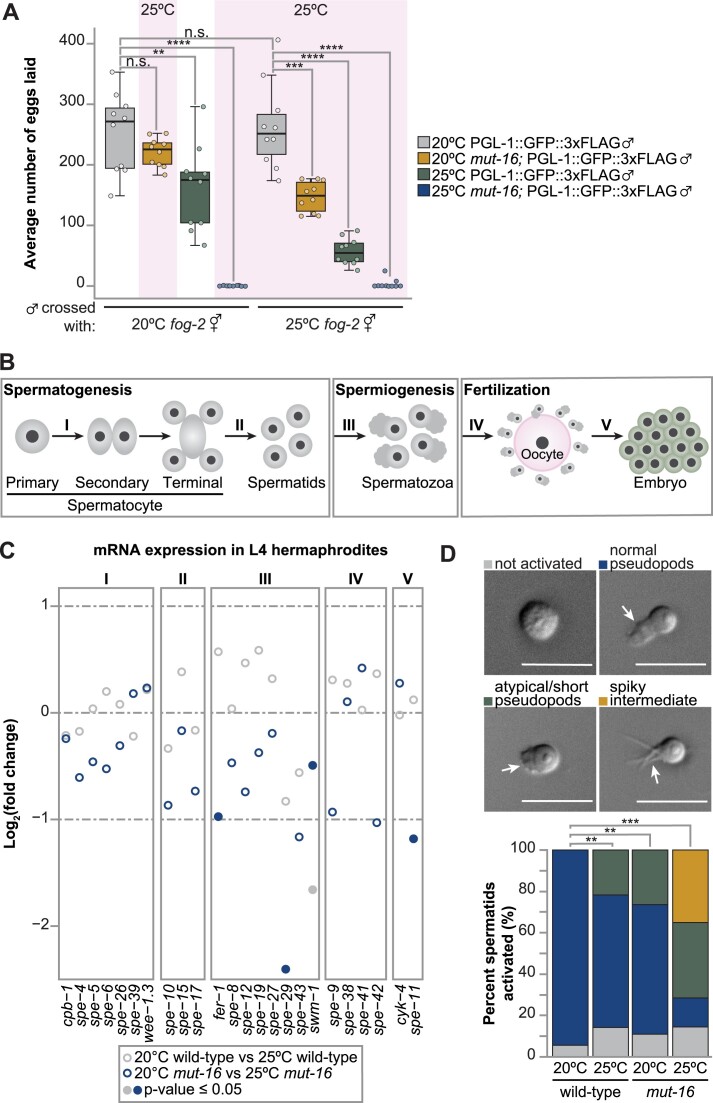
Spermiogenesis defects that trigger the onset of temperature-sensitive sperm-based sterility in *mut-16* mutants. (**A**) Box plots depicting average number of eggs laid for *fog-2(oz40*) hermaphrodites cultured at 20ºC or 25ºC mated with *pgl-1::GFP::3xFLAG* males grown at 20ºC (gray), *pgl-1::GFP::3xFLAG* males grown at 25ºC (gold), *mut-16(pk710); pgl-1::GFP::3xFLAG* males grown at 20ºC (green), or *mut-16(pk710); pgl-1::GFP::3xFLAG* males grown at 25ºC (blue). Circles represent number of GFP-expressing eggs counted for individual hermaphrodites after mating. Bolded midline indicates median value, box indicates the first and third quartiles, and whiskers represented the most extreme data points within 1.5 times the interquartile range, excluding outliers. *N* = 10. (**B**) Schema of stages of sperm development critical for male reproductive potential. I) primary spermatocyte division to form secondary spermatocytes, II) spermatid formation, III) spermatid activation during spermiogenesis, IV) spermatozoa enter the oocyte during fertilization, and V) post-fertilization viability of the embryo (**C**) Strip plot showing the change in expression of genes essential for each stage of sperm development (corresponding to schema in B). Circles represent the log_2_(fold change) for each gene, as determined by DESeq2, for comparisons between wild-type L4 hermaphrodites cultured at 20ºC and 25ºC (gray) and *mut-16* mutant L4 hermaphrodites cultured at 20ºC and 25ºC (blue). Filled circles indicate a significant *p* value as determined by DESeq2 (*P*-value ≤ 0.05). For each genotype and condition, two biological replicates were sequenced. (**D**) Shown are representative images of spermatids isolated from male adult animals after exposure to pronase E. White arrows indicate pseudopods and scale bars indicate 10 μm. Below, percent of pronase E-treated spermatids that are not activated (gray), have normal pseudopod formation (blue), atypical pseudopod formation (green), or arrest as spiky intermediates (gold) are shown for wild-type and *mut-16* adult males grown at 20ºC and 25ºC. n = 200 per genotype for each condition. For (A, D) two-tailed Welch's *t*-tests were performed to determine statistical significance. n.s. denotes not significant and indicates a *P*-value > 0.05, * indicates a *P*-value ≤ 0.05, ** indicates a *P*-value ≤ 0.01, *** indicates a *P*-value ≤ 0.001, and **** indicates a *P*-value ≤ 0.0001.

We next sought to identify the underlying physiological cause of sperm-based sterility in heat stressed *mut-16* mutants. Our previous work established that *mut-16(pk710)* mutant hermaphrodites exhibit both oocyte-based and sperm-based temperature-sensitive sterility (39). First, we confirmed *mut-16* mutant males exhibit heat stress-induced sterility. To this end, we mated *fog-2(oz40)* hermaphrodites, which are unable to produce their own sperm (9), with wild-type (N2) or *mut-16* mutant males cultured at 20°C and 25°C. Males carried the PGL-1::GFP::3xFLAG transgene to ensure only cross progeny were counted. Synchronized L1 animals were cultured at 20ºC or 25ºC temperature prior to mating plate set up, and crosses were maintained at 25ºC if either individual was raised at elevated temperature. Indeed, *mut-16* mutant males cultured at 25ºC were unsuccessful in producing progeny after mating, regardless of the temperature at which the mated hermaphrodite was grown (Figure 6A). This corroborates our previous observation that the sperm-based effect of *mut-16* manifests after a single generation of elevated temperature (39), and indicates that the reproductive potential of both *mut-16* mutant hermaphrodite- and male-derived sperm is immediately impacted.

Male infertility can arise from changes in male mating behavior, defects in somatic tissues required for spicule insertion and sperm transfer, or abnormalities in sperm production and/or activation. We did not identify severe morphological defects in the male germlines or post-meiotic spermatid counts (Supplementary Figure S5A and B). Next, we assessed the morphology of the somatic spicule tissue of males, which is critical for probing the vulva and facilitating sperm transfer during mating. Previously, in wild-type male *C. elegans*, it was shown that heat stress results in shortened spicule length and increased incidence of malformed or crumpled spicules (62). Indeed, we observed overall shorter average spicule lengths and increased rates of malformed/crumpled spicules in wild-type males grown at 25ºC compared to those grown at 20ºC (Supplementary Figure S5C and D). At permissive temperature (20ºC), the *mut-16* mutation resulted in an overall shorter average spicule length compared to wild-type animals, but not increased incidence of crumpled spicules. The shortening of the average spicule length was exacerbated in heat-stressed *mut-16* mutant males, which also exhibited increased rates of malformed/crumpled spicules (Supplementary Figure S5C and D). Overall, our data indicates that the underlying cause of temperature-sensitive sterility in *mut-16* mutant males is not germline tissue abnormalities or reduced production of spermatids. Heat stress and the *mut-16* mutation do trigger mild defects in the male somatic tissues that facilitate sperm transfer during mating; however, as these defects are not fully penetrant in the population, they are not sufficient to explain the temperature-sensitive sterility of *mut-16* mutant males.

We next aimed to determine if loss of sperm reproductive potential in heat stressed *mut-16* mutants occurs due to defects in spermatogenesis, spermiogenesis, or during fertilization. We assessed the changes in expression of genes critical for proper completion of I) primary spermatocyte division to form secondary spermatocytes, II) spermatid formation, III) spermatid activation during spermiogenesis, IV) spermatozoa entering the oocyte during fertilization, and V) post-fertilization viability of the embryo (Figure 6B). In agreement with the presence of abundant post-meiotic spermatids in the gonad of heat stressed *mut-16* mutant males, we found genes critical for proper division of spermatocytes and spermatid formation are mildly, but not statistically significantly (*P*-value ≤ 0.05), down-regulated in heat stressed *mut-16* mutant L4 hermaphrodites (Figure 6C). Spermatids transform into motile spermatozoa through the process of spermiogenesis, during which exposure to an activator causes a spermatid to extend long spikes that are restructured to form a pseudopod (63,64). Our differential expression analysis revealed that all the genes essential for proper spermiogenesis had reduced expression in heat stressed *mut-16* mutant L4 hermaphrodites, but only *fer-1, spe-29*, and *swm-1* were statistically significantly down-regulated (Figure 6C). It should be noted that *swm-1* was also significantly down-regulated at 25ºC in wild-type animals, indicating expression of *swm-1* is particularly sensitive to heat stress (Figure 6C). Loss of functional FER-1 triggers formation of short, nonmotile pseudopods during spermiogenesis (65–68), whereas spermatids in *swm-1* mutants prematurely activate prior to mating (69). Mutations in the *spe-8* pathway (*spe-8*, *spe-12*, *spe-27*, and *spe-29*), which is critical for *in vivo* and *in vitro* spermatid activation, result in spermatozoa arresting as nonmotile, spiky intermediates (25,63,64). Our analysis of the genes required for fertilization and post-fertilization viability of the embryo found that only *spe-11* was significantly differentially expressed in heat stressed *mut-16* mutant L4 hermaphrodites (Figure 6C). Spermatozoa lacking *spe-11* can fertilize oocytes to produce embryos; however, after these eggs are laid, embryogenesis fails, resulting in paternal-effect embryonic lethality (64). In our mating brood size assay, *fog-2* hermaphrodites crossed to heat stressed *mut-16* mutants males did not lay fertilized eggs (Figure 6A). Thus *spe-11*-induced paternal-effect embryonic lethality is not likely to be the driving force of temperature-sensitive sperm-based sterility in *mut-16* mutant animals.

Our bioinformatics analyses pointed to spermiogenesis defects underlying heat stress-induced loss of sperm-based fertility in *mut-16* mutants. To test this, we performed an *in vitro* activation assay on spermatids obtained from wild-type and *mut-16* mutant males grown to adulthood at 20ºC and 25ºC. We assessed pseudopod formation after exposure to the activation agent, pronase E, and classified pseudopod formation as normal, atypical (short or two distinct pseudopods), or as spiky intermediates (Figure 6D). At permissive temperature (20ºC), wild-type spermatids had a 95% activation rate, with all pseudopods forming normally. Heat stress lowered the activation rate of wild-type spermatids to 86% and increased the incidence of atypical pseudopod formation (22% short pseudopods) (Figure 6D). Spermatids collected from *mut-16* mutant males at 20ºC exhibited similar rates of activation (89%) and incidence of atypical pseudopod formation (27%) as the heat-stressed wild-type spermatids (Figure 6D). Strikingly, spermatids collected from *mut-16* males grown at 25ºC had an 86% activation rate, however the majority of the activated sperm formed atypical pseudopods or arrested as spiky intermediates (37% and 35%, respectively) (Figure 6D). These data indicate that heat stress and the *mut-16* mutation independently trigger comparable spermiogenesis defects, but the combination of both leads to severely compromised pseudopod formation that ultimately drives the temperature-sensitive loss of sperm-based fertility in *mut-16* mutants.

## Discussion

**Figure 7.**
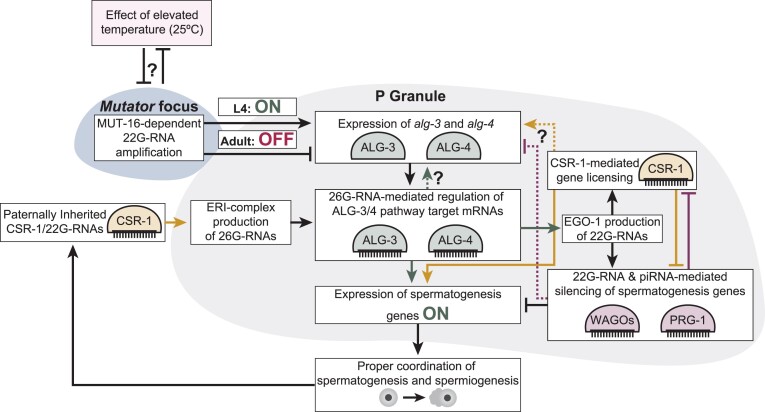
MUT-16-dependent small RNAs are required to restrict ALG-3/4 pathway function to the L4 developmental stage. Model of the small RNA-mediated regulatory network in which MUT-16-dependent 22G-RNAs are required to counterbalance the effects of heat stress and control expression of *alg-3* and *alg-4* to maintain developmental stage-specific ALG-3/4 pathway (green) function that, with the CSR-1 pathway (yellow) and piRNA pathway (purple), regulates expression of spermatogenesis genes to maintain sperm-based fertility. Dashed lines with question marks indicate hypothetical regulation of *alg-3* and *alg-4* by different RNAi pathways.

Evolutionarily conserved RNAi pathways serve as one gene regulatory mechanism essential for robust coordination of cellular processes in all animals. When the gene regulatory networks controlling physiological processes are disrupted by genetic or environmental stressors, severe consequences can arise, such as loss of reproductive potential. However, the regulatory motifs that coordinate RNAi pathways are not well characterized. Here, we show RNAi pathways that use *mutator* complex amplification contribute to developmental stage-specific regulation of the ALG-3/4 pathway through small RNA-mediated gene switches that turn off and on the expression of *alg-3* and *alg-4*. Proper execution of the genetic switches is critical for thermotolerant sperm-based fertility and mitigating the effects of heat stress, which negatively impacts gametogenesis across animal species (70–72). We propose a model in which *mutator*-dependent 22G-RNA amplification is required for RNAi pathways to engage the genetic switches that promote the expression of *alg-3* and *alg-4* during the L4 stage, possibly in concert with autoregulatory feedback from ALG-3/4-bound 26G-RNAs. This RNAi-mediated regulation of the ALG-3/4 AGOs would in turn coordinate ALG-3/4 pathway positive and negative regulation of sperm transcripts to mitigate the effects of heat stress and maintain robust sperm-based fertility. Then, during the adult stage, changes in the pool of *mutator* complex-dependent 22G-RNAs would drive the genetic cascade that switches off *alg-3* and *alg-4* expression. Ultimately, the RNAi-to-RNAi cascade regulating *alg-3* and *alg-4* expression would provide robustness to the spermatogenesis developmental program during heat stress (Figure 7). But how does small RNA-targeting promote the expression of *alg-3* and *alg-4* instead of silence it? One possibility is that targeting of *alg-3* and *alg-4* transcripts by one RNAi branch protects them from degradation by another. If this is the case, the loss of different classes of small RNAs in the heat-stressed *mut-16* mutants could disrupt the balance of the antagonism, rendering the *alg-3* and *alg-4* transcripts susceptible to degradation by various RNAi pathways. In fact, such antagonistic behavior between the RNAi branches has been previously proposed for the CSR-1 and PRG-1 pathways (34). CSR-1-targeting is required for maintaining active transcription of sperm-specific genes during the L4 stage (32), however, recent work indicates *alg-3* and *alg-4* are not targets of CSR-1-loaded 22G-RNAs (73). Perhaps another AGO could act to protect *alg-3* and *alg-4* transcripts from RNAi-mediated silencing. Future work examining the possibility of antagonism between 22G-RNA and 26G-RNA pathways targeting *alg-3* and *alg-4* will be critical for our understanding of how homeostasis is maintained between multiple overlapping RNAi pathways to ensure appropriate regulation of target transcripts.

Our model postulates that MUT-16 acts upstream of ALG-3/4 pathway function in maintaining appropriate spermatogenesis gene expression by small RNA-mediated regulation of *alg-3* and *alg-4* expression. If this is the case, we would expect *mut-16* mutants and *alg-3; alg-4* mutants to phenocopy each other. Previous work showed that ALG-3 and ALG-4 function redundantly to provide thermotolerant sperm-based fertility, and that double *alg-3; alg-4* mutants exhibit ∼50% reduced fertility compared to wild-type animals at 20ºC and complete sterility when cultured at elevated temperature (25,32). During the L4 stage, we see reduced expression of both *alg-3* and *alg-4* in *mut-16* mutants grown at permissive temperature (20ºC) compared to wild-type L4 animals. In addition, we have shown here and previously that *mut-16* mutants have ∼50% fewer progeny compared to wild-type animals when cultured at 20ºC and exhibit complete sterility when cultured at 25ºC (39). The comparable fertility phenotypes is suggestive of a common defect in sperm-based fertility in *mut-16* and *alg-3; alg-4* mutants. Indeed, spermatids of heat-stressed *alg-3; alg-4* mutants exhibit spermiogenesis defects, specifically arresting as spiky intermediates upon pronase E activation (25,32), comparable to the incidence of spermatids that arrest as spiky intermediates at the onset of temperature-sensitive sterility in *mut-16* mutants. Here, we show that heat stress causes a severe loss of *alg-3* and *alg-4* expression in *mut-16* mutants, that coincides with the spermiogenesis defects. Taken together, these results indicate *mut-16* and *alg-3; alg-4* mutants have analogous thermosensitive sperm-based infertility phenotypes. Furthermore, the *mutator* complex is intact, and thus amplification of *mutator-*dependent 22G-RNAs is not disrupted, in the *alg-3; alg-4* mutants, suggesting that down-regulation of *alg-3* and *alg-4* in the *mut-16* mutants is sufficient to induce the heat-sensitive sterility triggered by the observed spermiogenesis defects.

The ALG-3 and ALG-4 Argonautes bind 26G-RNAs, which are not synthesized by the *mutator* complex, so how does loss of *mut-16* trigger reduced 26G-RNA levels targeting ALG-3/4 pathway targets? Canonically, the production of 22G-RNAs is downstream of 26G-RNA biogenesis and relies on recognition of target transcripts by 26G-RNA-loaded RISC. Thus, a feedback loop is required for changes in 22G-RNA biogenesis to impact upstream 26G-RNA levels. In fact, a feedforward mechanism was previously proposed in which CSR-1-loaded 22G-RNAs packaged in sperm initiates production of 26G-RNAs that can be loaded into ALG-3/4 within the next generation's germline. Then ALG-3/4 recognition of sperm-enriched transcripts results in amplification of 22G-RNAs that can be loaded into CSR-1 and packaged in new sperm (32). However, this feedback mechanism does not explain how ALG-3/4-loaded 26G-RNAs are impacted by loss of MUT-16 as CSR-1-class 22G-RNAs do rely on *mutator* complex-dependent amplification. Previously, we identified a small RNA-mediated feedback loop in which MUT-16-dependent 22G-RNAs regulate the expression of the ERGO-1-class 26G-RNA biogenesis factor, ERI-6/7 (60). In this feedback mechanism, loss of MUT-16-dependent 22G-RNAs triggers reduced expression of trans-spliced *eri-6/7* mRNAs, thereby indirectly regulating the upstream production of ERGO-1-class 26G-RNAs (60). We hypothesize the loss of 26G-RNAs mapping to ALG-3/4 pathway targets, as well as *alg-3* and *alg-4*, observed in the *mut-16* mutant at 25ºC is the result of another small RNA-mediated feedback loop. Reduced ALG-3/4 protein levels due to loss of 22G-RNAs targeting *alg-3/4* mRNA levels could indirectly trigger reduced production and/or stability of 26G-RNAs mapping to ALG-3/4 pathway targets. Whether the small RNAs mapping to *alg-3* and *alg-4* act in autoregulation by the ALG-3/4 and CSR-1 pathway or direct regulation by one, or multiple, other RNAi pathway branches remains to be explored. Furthermore, our mRNA-seq and small RNA-seq data revealed the small RNAs that act to regulate the expression of *alg-3* and *alg-4* are thermosensitive. This putative small RNA-mediated feedback mechanism warrants additional exploration to determine how it modulates *alg-3* and *alg-4* expression in response to changes in environmental temperature and fluctuations in small RNA populations, and to further elucidate the complexities of the RNAi pathways that regulate developmental cellular programs. Moving forward, identifying the molecular mechanism(s) underlying thermosensitivity of small RNA populations will be critical for advancing our understanding of how appropriate gene expression is maintained across developmental time in the face of environmental perturbations.

As a consequence of the dysregulation of the ALG-3/4 pathway at elevated temperature in *mut-16* mutants, we show sperm gene expression is also developmentally dysregulated. This finding implicates the *Mutator* focus and *mutator* complex-dependent 22G-RNA amplification in coordinating the spermatogenesis developmental program. Coordination of initiation and termination of the spermatogenesis and oogenesis developmental programs is critical for maintaining the reproductive potential of *C. elegans*. Typically, in hermaphroditic *C. elegans*, the spermatogenesis and oogenesis programs occur sequentially during discrete developmental timeframes within the gonad (74). Our bioinformatic analyses revealed that loss of MUT-16-dependent 22G-RNA amplification triggers the developmental dysregulation of the spermatogenesis program, but largely does not impact the oogenesis program at the mRNA level. In addition, we found that when RNAi is disrupted, expression of sperm genes is more susceptible to perturbation by heat stress than that of oogenesis genes. Our previous work, coupled with our findings here, suggest that spermatogenesis- and oogenesis-enriched genes are controlled by distinct layers of regulatory mechanisms. Furthermore, our data points to the spermatogenesis- and oogenesis-enriched genes being independently coordinated such that expression of oogenesis genes does not rely on the termination of the spermatogenesis developmental program. These findings have broad implications for our understanding of the processivity and regulation of developmental programs in germ cells. For example, if the spermatogenesis and oogenesis programs can act on the same pool of germ cells at the same time, what drives the cell fate decision for each germ cell in the gonad? A substantial amount of work has mechanistically dissected the sex determination pathway in the germline of *C. elegans*. This work revealed that post-transcriptional regulatory modes play a role in the spermatogenesis-to-oogenesis switch by maintaining specific ratios of TRA-2 to FEM-3, which is a critical determinant of whether germ cells become sperm or oocytes (9–10,15–16,75). However, it remains unclear what coordinates these regulatory modes to ensure changes in the TRA-2 to FEM-3 ratios at specific developmental time points to enable sperm production followed by oocyte formation in hermaphrodites. Based on our findings, perhaps RNAi pathways play a role in maintaining robust coordination of the post-transcriptional regulatory modes that dictate the spermatogenesis-to-oogenesis switch. Future work will be needed to determine if RNAi pathways safeguard germ cell fate during heat stress by maintaining homeostatic *tra-2* and *fem-3* transcript levels, regulating the expression of factors that dictate the TRA-2 to FEM-3 ratio, or regulating other pathways that influence germ cell fate.

For sperm-based fertility, transcription of spermatogenesis genes is necessary during the narrow L4 developmental timeframe but must be suppressed during the adult developmental stage for proper oocyte-based fertility. Previous works have determined that appropriate regulation of spermatogenesis genes requires the ALG-3/4, CSR-1 and PRG-1 pathways, which localize to P granules – perinuclear germ granules that sit adjacent to *Mutator* foci (25,29,30,32,34–38,42,54,76–81). P granules are hallmarks of germ cell identity and persist in oocytes but are disassembled in spermatids during spermiogenesis, thus their presence within the adult germline is indicative of successful completion of the spermatogenesis to oogenesis program switch (82–84). Here we show that the absence of MUT-16-dependent small RNAs results in reduced spermatogenesis-enriched gene expression during the L4 stage and aberrant up-regulation of sperm genes in adults. This phenomenon is observed in *mut-16* mutants cultured at permissive temperature (20ºC) but is more pronounced at elevated temperature (25ºC) when the animals exhibit temperature-sensitive sterility. Delayed spermatogenesis in *mut-16* mutants experiencing heat stress could have sex-specific effects. In hermaphrodites, the mis-regulation of spermatogenesis could trigger a delay in the spermatogenesis-to-oogenesis switch, whereas in males the mis-regulation of spermatogenesis could result in prolonged production of post-meiotic spermatids. Indeed, we observed slightly increased numbers of post-meiotic spermatids in male *mut-16* mutants grown at elevated temperature. Moreover, heat-stressed hermaphroditic adult *mut-16* animals that should be undergoing oogenesis have aberrant expression of spermatogenesis-enriched genes, coupled with P granule abnormalities in their germ cells (39), suggesting a defect in the switch between the spermatogenesis and oogenesis programs. A similar pattern of reduced spermatogenesis gene expression during the L4 developmental stage followed by sperm gene up-regulation during adulthood has been previously observed in *prg-1* mutants, which have a defective piRNA pathway (42,85). Sterility in piRNA pathway mutants also correlates with abnormalities in *Mutator* foci and P granule formation (86). In addition, *csr-1* mutants, which have an aggregated P granule phenotype, and *C. elegans* that have been fed RNAi for *pgl-1, pgl-3, glh-1* and *glh-4*, and thus completely lack P granules, also exhibit increased expression of spermatogenesis genes in adults resulting in masculinization of the adult hermaphroditic germline (38). Taken together, these findings suggest that *Mutator* foci and P granules coordinate the PRG-1, CSR-1 and ALG-3/4 pathways to properly initiate and terminate the spermatogenesis developmental program.

Previously, a model was proposed in which P granules and the Argonaute, CSR-1, work together to coordinate the spermatogenesis-to-oogenesis switch through small RNA-mediated suppression of spermatogenesis-enriched gene transcript levels in germ cells at the beginning of oogenesis (38). This model builds on foundational work that discovered ALG-3 and ALG-4 bind 26G-RNAs complementary to sperm-enriched genes, and are expressed during spermatogenesis (L4 stage) to positively and negatively regulate sperm gene transcripts to maintain homeostatic mRNA levels to provide thermotolerant sperm-based fertility in males and hermaphrodites (25). Many ALG-3/4 pathway and CSR-1 pathway targets overlap, so it was proposed that regulation of targets by the ALG-3/4 pathway during the L4 stage triggers MUT-16-independent 22G-RNA production from sperm gene mRNAs for downstream loading into CSR-1 and WAGO-1 (32). These CSR-1-loaded 22G-RNAs are thought to then act in a transgenerational feedforward loop providing sperm-mediated transmission of the epigenetic memory of gene expression necessary for the next generation's fertility (32). These models relied on L4 stage-specific expression of ALG-3 and ALG-4 driving ALG-3/4 pathway function to appropriately regulate spermatogenesis-enriched genes; yet how temporal regulation of these Argonautes is achieved remained elusive until now. This work has identified a gene regulatory network architecture in which an RNAi to RNAi-mediated cascade regulates the function of an RNAi pathway branch to ensure homeostasis of a developmental program during stress. RNAi pathway-mediated gene regulation is critical for the viability of all animals, and there is exciting potential for more RNAi-mediated feedback network architectures that coordinate cellular programs in eukaryotes to be discovered in the future.

## Supplementary Material

gkae586_Supplemental_Files

## Data Availability

High-throughput sequencing data for mRNA-seq and small RNA-seq experiments generated during this study are available through Gene Expression Omnibus (GEO #: GSE226893). De-multiplexed raw sequencing data, in fastq format, for N2 and *mut-16(pk710)* adult mRNA-seq and small RNA-seq libraries used from (39) were obtained from NCBI’s Gene Expression Omnibus (GEO #: GSE134573). Source data are provided with this paper.
