# Supplementary material for: Small RNA-mediated genetic switches coordinate ALG-3/4 small RNA pathway function
Source: Nucleic Acids Res. 2024 Jul 5;52(16):9431–49. doi: 10.1093/nar/gkae586 (PMC11381353; doi:10.1093/nar/gkae586)
Supplement: gkae586_Supplemental_Files [file gkae586_supplemental_files.zip › Sen_McCormick_and_Rogers_Supplementary_Information.pdf]

## SUPPLEMENTARY FIGURES AND FIGURE LEGENDS

## SUPPLEMENTARY FIGURE S1

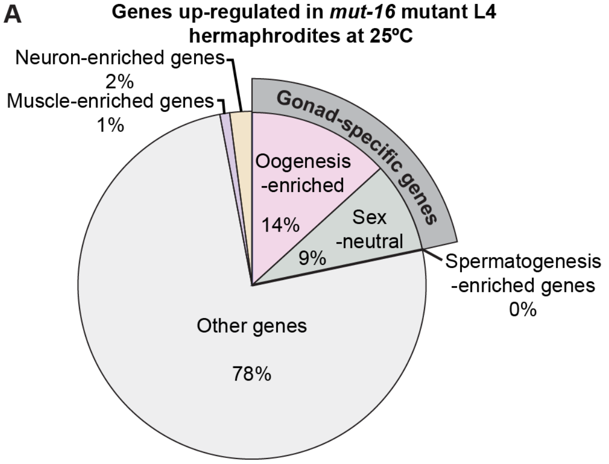


**B** **Enrichment analysis of genes up-regulated in *mut-16* mutant L4 hermaphrodites at 25ºC**

| Gene lists | $log_2$(enrichment) | Significance |
|---|---|---|
| Spermatogenesis-enriched genes | -INF | *** |
| Oogenesis-enriched genes | 2.64 | **** |
| Sex-neutral gonad genes | 1.72 | **** |
| Muscle & neuronal-specific genes | -0.33 | n.s. |

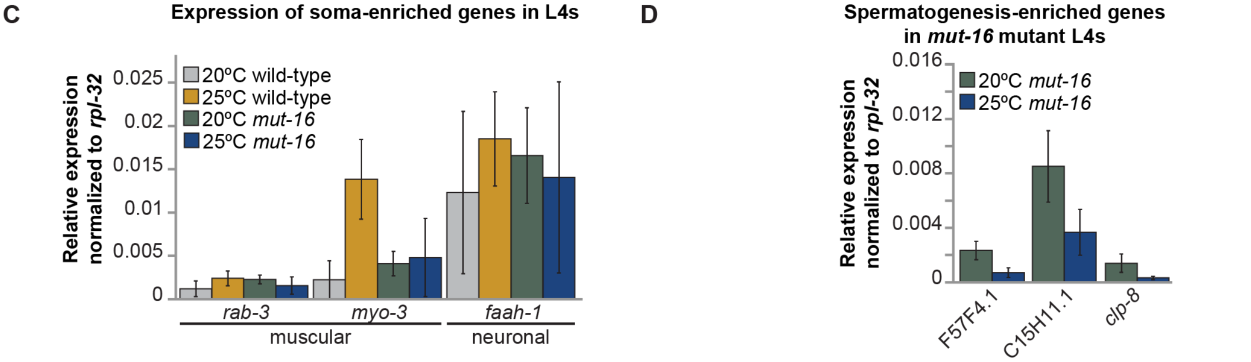


**Supplemental Figure S1. (A)** Percentages of gonad-specific and non-gonad-specific genes represented in the genes up-regulated exclusively in *mut-16* mutant L4s at 25°C. **(B)** Enrichment analysis for spermatogenesis, oogenesis, sex-neutral genes, and muscle-specific and neuronal-specific genes amongst the genes up-regulated during heat stress in *mut-16* mutants is shown. Two-tailed *p* values for enrichment or depletion were calculated using the Fisher's exact test function in R. -INF indicates there were no up-regulated genes overlapping with the gene list. n.s. denotes not significant and indicates a p-value > 0.05, *** indicates a p-value ≤ 0.001, and **** indicates a p-value ≤ 0.0001. **(A, B)** For each genotype and condition, two biological replicates were sequenced. **(C)** qRT-PCR for genes expressed in muscle and neuronal cells in wild-type and *mut-16* mutant L4 hermaphrodites grown at 20ºC and 25ºC, with bar graphs representing the mean and error bars indicating standard deviation. Expression is normalized to *rpl-32*. n = 4 biological replicates. **(D)** qRT-PCR of spermatogenesis-enriched genes (previously observed to be up-regulated in the germline of adult *mut-16* mutant hermaphrodites at 25ºC[25]) in *mut-16* mutant L4 hermaphrodites grown at 20ºC and 25ºC, with bar graphs representing the mean and error bars indicating standard deviation. Expression is normalized to *rpl-32*. n = 4 biological replicates.

## SUPPLEMENTARY FIGURE S2

A

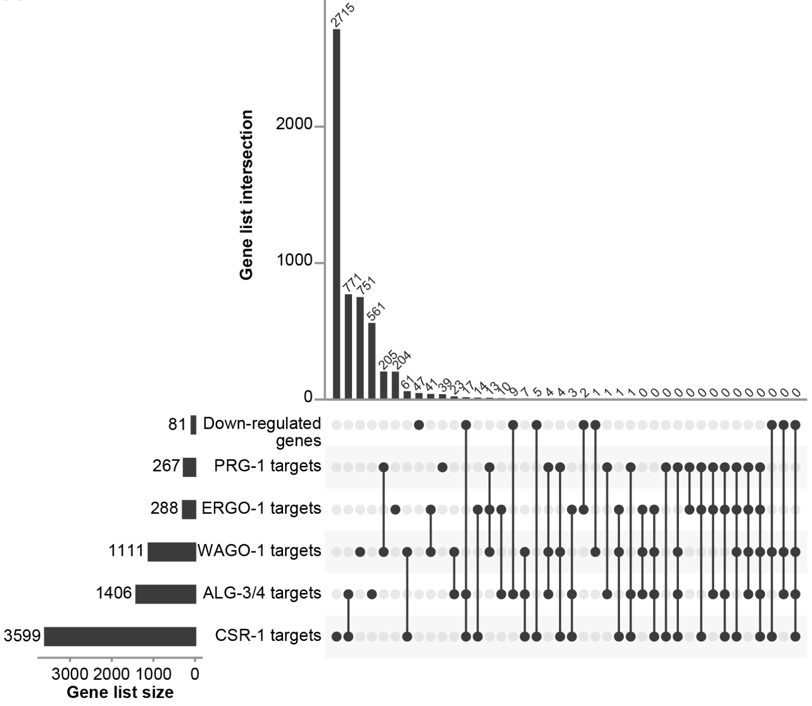


B

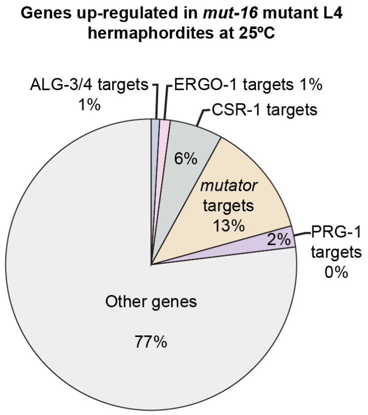


C

**Enrichment analysis of small RNA targets for genes up-regulated in *mut-16* mutants at 25ºC**

| Gene lists | $log_2$(enrichment) | Significance |
|---|---|---|
| ALG-3/4 targets | -1.69 | ** |
| ERGO-1 targets | 0.92 | n.s. |
| CSR-1 targets | -0.40 | n.s. |
| *mutator* targets | 1.57 | **** |
| PRG-1 targets | 2.04 | *** |

**Supplementary Figure S2. (A)** Upset plot depicting the overlap of genes targeted by distinct small RNA pathways (ALG-3/4, ERGO-1, CSR-1, WAGO-1, and PRG-1 pathways) and the genes down-regulated exclusively in *mut-16* mutant L4 hermaphrodites at 25°C. **(B)** Percentage of genes targeted by distinct small RNA pathways (ALG-3/4, ERGO-1, CSR-1, *mutator*, and PRG-1 pathways) represented in the genes up-regulated exclusively in *mut-16* mutant L4 hermaphrodites at 25°C. **(C)** Enrichment analysis for ALG-3/4, ERGO-1, CSR-1, *mutator*, and PRG-1 pathway targets amongst the genes up-regulated during heat stress in *mut-16* mutants is shown. Two-tailed *p* values for enrichment was calculated using the Fisher's exact test function in R. n.s. denotes not significant and indicates a p-value > 0.05, ** indicates a p-value ≤ 0.01, *** indicates a p-value ≤ 0.001, and **** indicates a p-value ≤ 0.0001. **(B, C)** For each genotype and condition, two biological replicates were sequenced.

## SUPPLEMENTARY FIGURE S3

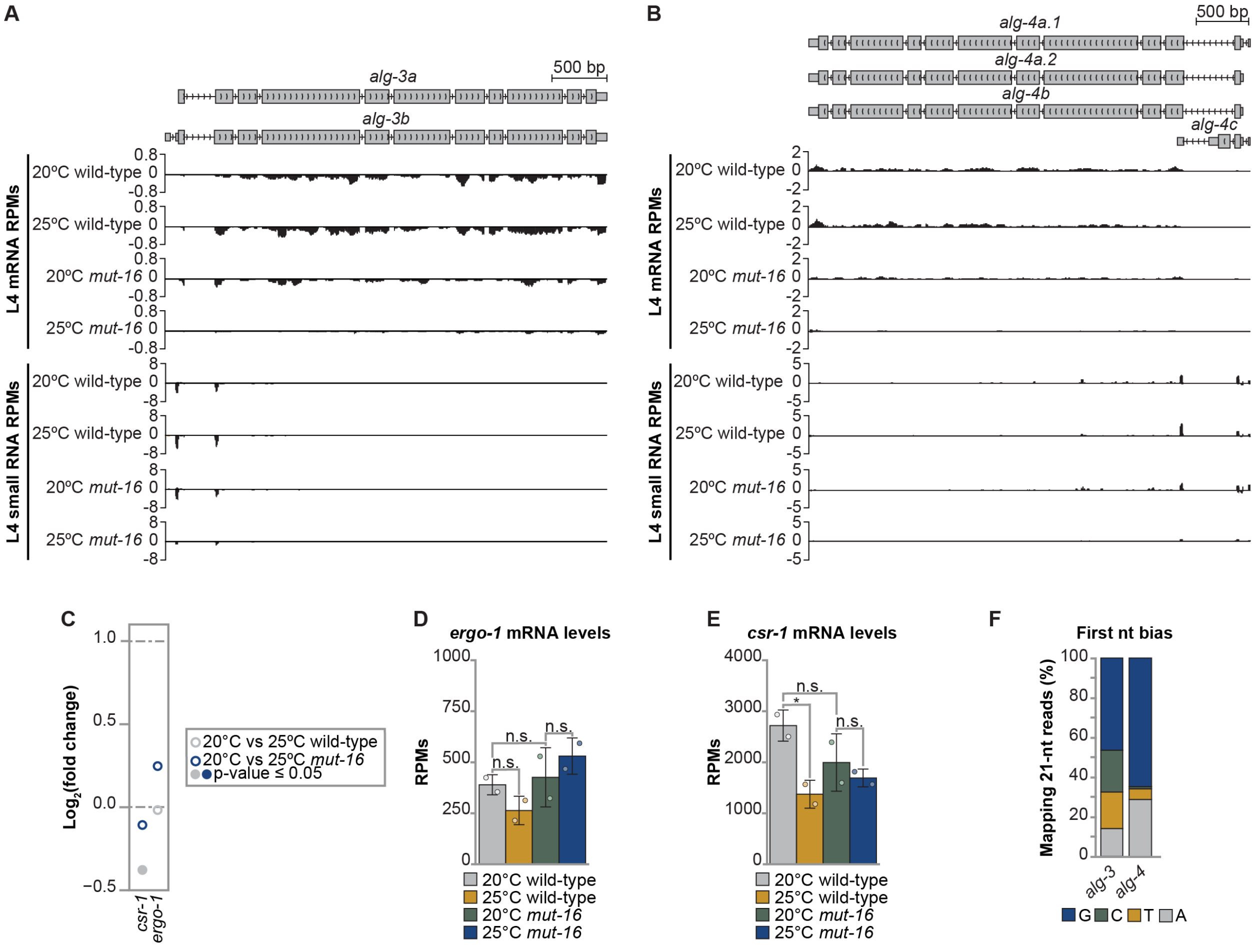


**Supplementary Figure S3.** Representative tracks shown for mRNA and small RNA reads per million (RPMs) mapping to the **(A)** *alg-3* and **(B)** *alg-4* genomic loci in wild-type and *mut-16* mutant L4 hermaphrodites cultured at 20°C and 25°C. **(C)** Strip plot showing the change in expression of *csr-1* and *ergo-1*. Circles represent the $\log_2$(fold change) for each gene, as determined by DESeq2, for comparisons between wild-type L4 hermaphrodites cultured at 20ºC and 25ºC (gray) and *mut-16* mutant L4 hermaphrodites cultured at 20ºC and 25ºC (blue). Filled circles indicate a significant *p* value as determined by DESeq2 (p-value ≤ 0.05). mRNA reads mapping to **(D)** *egro-1* and **(E)**.*csr-1* are counted, in reads per million (RPMs), for wild-type and *mut-16* mutant L4 animals cultured at 20°C and 25°C. **(D, E)** Bar graphs represent the mean with dots representing summed RPMs for biological replicates and error bars indicating standard deviation. Two-tail Welch's *t*-tests were performed to determine statistical significance. n.s. denotes not significant and indicates a p-value > 0.05, * indicates a p-value ≤ 0.05. **(F)** Shown is the percentage of 21-nt reads mapping to *alg-3* and *alg-4* with A, T, C, or G represented in the first position of the read in wild-type L4 hermaphrodites cultured at 20ºC

## SUPPLEMENTARY FIGURE S4

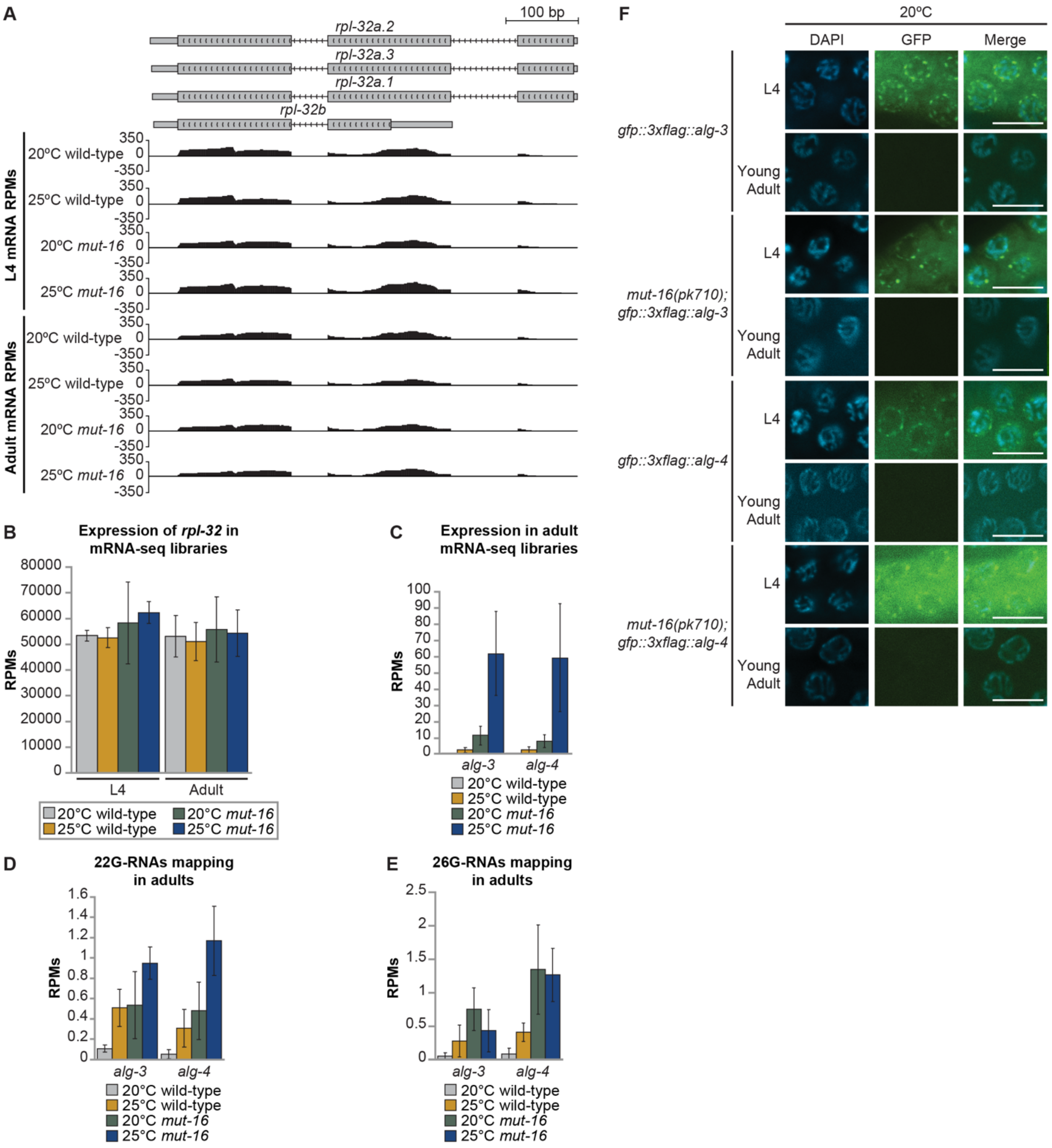


**Supplementary Figure S4. (A)** Representative track of mRNA reads per million (RPMs) mapping to the *rpl-32* genomic locus in wild-type and *mut-16* mutant L4 and adult hermaphrodites cultured at 20°C and 25°C. **(B)** mRNA reads mapping to *rpl-32* are counted, in reads per million (RPMs), for wild-type and *mut-16* mutant L4 and adult hermaphrodites cultured at 20°C and 25°C. **(C)** mRNA transcripts mapping to *alg-3* and *alg-4* are counted, in reads per million (RPMs), for wild-type and *mut-16* mutant adult hermaphrodites cultured at 20°C and 25°C. **(D)** 22G-RNAs and **(E)** 26G-RNAs mapping to *alg-3* and *alg-4* are counted, in reads per million (RPMs), for wild-type and *mut-16* mutant adult hermaphrodites cultured at 20°C and 25°C. **(F)** Representative fluorescence microscopy images of germline nuclei from the proximal end of gonads of L4 and young adult stage animals expressing GFP::3XFLAG::ALG-3 or GFP::3XFLAG::ALG-4 in the wild-

type and *mut-16* mutant background grown at 20ºC. Scale bars indicate 10 μm. **(B, C, D, E)** Bar graphs represent the mean and error bars indicating standard deviation. **(A, B, C, D, E)** For each genotype and condition, two biological replicates were sequenced for L4-stage animals and three biological replicates were sequenced for adult-stage animals.

## SUPPLEMENTARY FIGURE S5

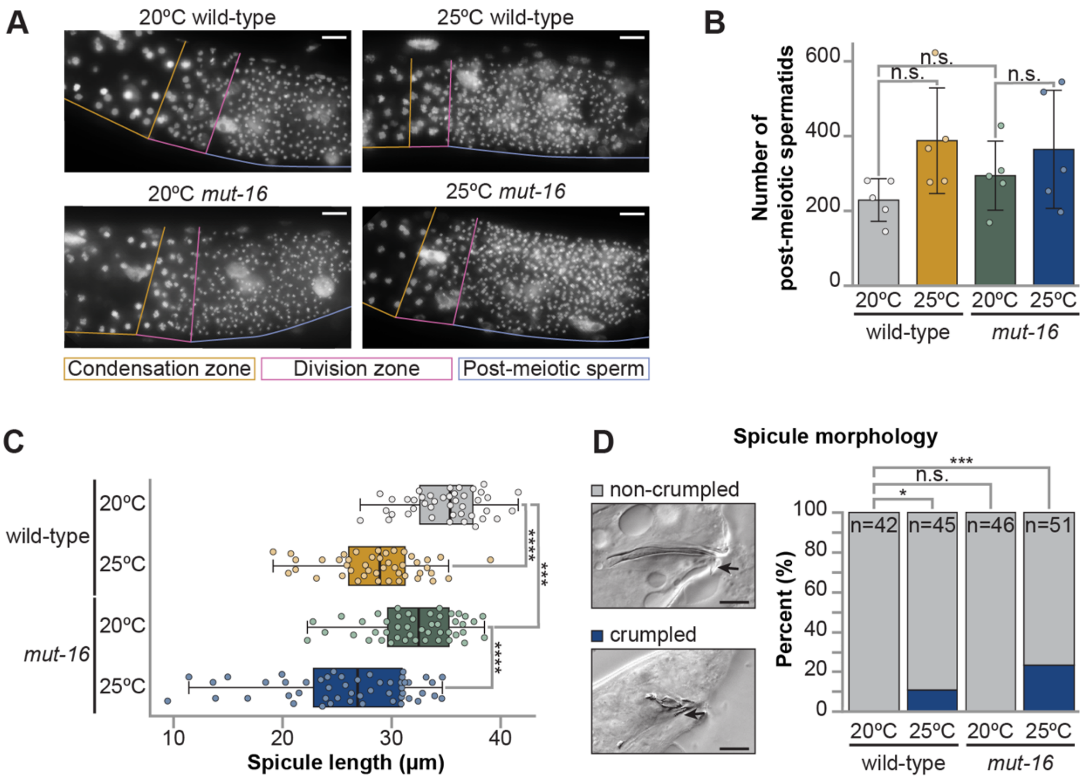


**Supplementary Figure S5. (A)** DAPI-stained nuclei in adult male gonads of wild-type and *mut-16(pk710)* animals cultured at 20ºC or 25ºC. Regions of the gonad are labeled: condensation zone (gold), meiotic division zone (pink), and haploid spermatids (blue). Five individual gonads were imaged for each genotype per condition. Scale bar indicates 10 µm. **(B)** Number of post-meiotic haploid spermatids in five male gonads of wild-type and *mut-16(pk710)* animals cultured at 20ºC or 25ºC are shown, with bar graphs representing the mean and error bars indicating standard deviation. **(C)** Box plots depicting average spicule length, in µm, measured for wild-type males grown at 20ºC (n = 42), wild-type males grown at 25ºC (n = 45), *mut-16* mutant males grown at 20ºC (n = 46), and *mut-16* mutant males grown at 25ºC (n = 51). Circles represent each spicule measured. Bolded midline indicates median value, box indicates the first and third quartiles, and whiskers represented the most extreme data points within 1.5 times the interquartile range, excluding outliers. **(D)** Bar graphs depicting percentage of spicules that were normal/non-crumpled (gray) or crumpled (blue) for wild-type and *mut-16(pk710)* males grown at 20ºC or 25ºC. At least 42 spicules were assessed for each genotype per condition. Representative images of non-crumpled and crumpled spicules are shown. Black arrows indicate the tip of the measured spicule. Scale bar indicates 10 µm. For **(B, C, D)** two-tail Welch's *t*-tests were performed to determine statistical significance. n.s. denotes not significant and indicates a p-value > 0.05, * indicates a p-value ≤ 0.05, *** indicates a p-value ≤ 0.001, and **** indicates a p-value ≤ 0.0001.

## SUPPLEMENTARY FIGURE S6

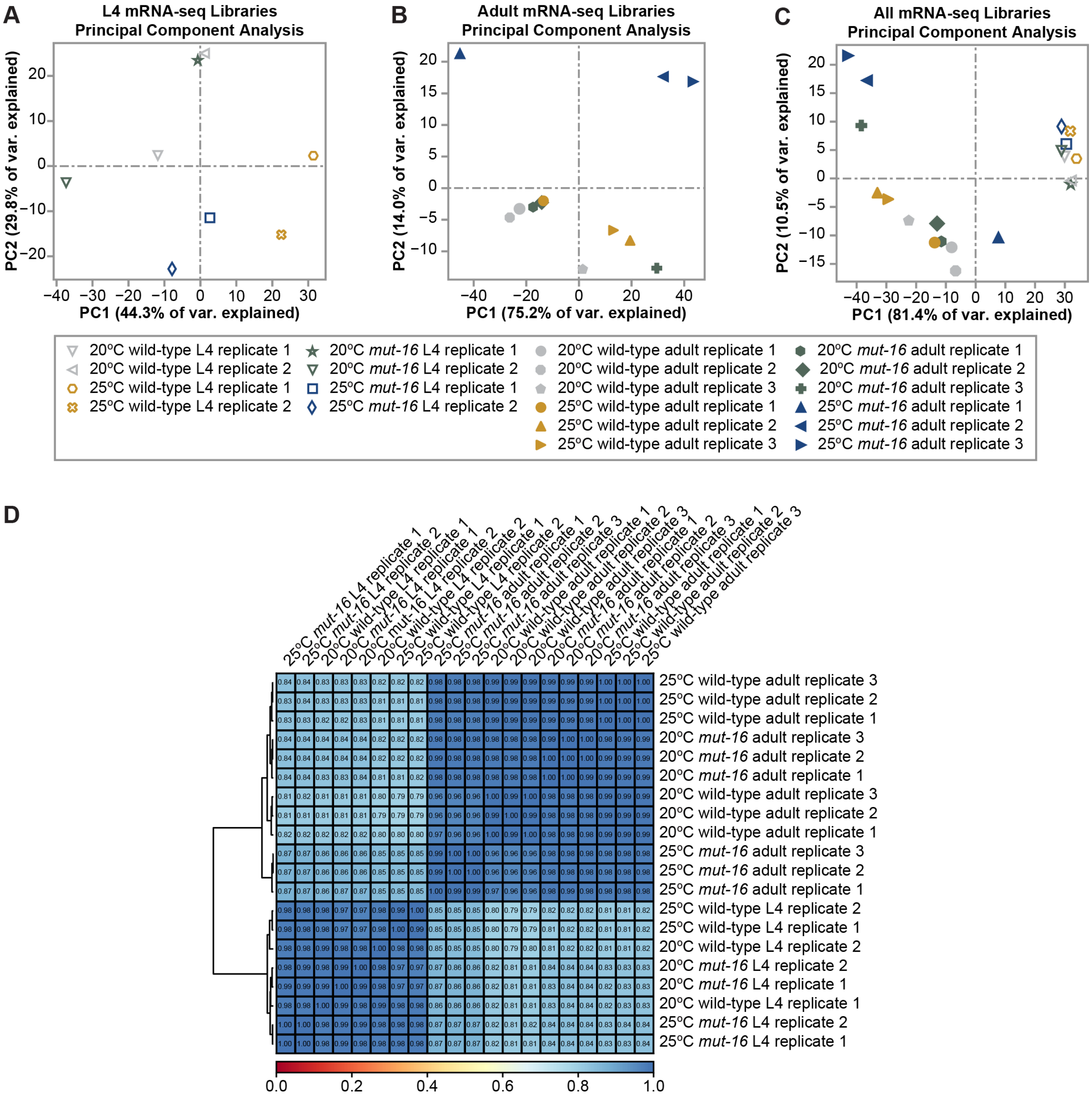


**Supplementary Figure S6.** Principal Component Analysis plots for **(A)** L4 mRNA-seq libraries, **(B)** adult mRNA-seq libraries, and **(C)** all mRNA-seq libraries. The key for the Principal Component Analysis plot symbols is shown below the plots. **(D)** Hierarchical clustering of global correlation coefficients between samples heat map for all mRNA-seq libraries.

## SUPPLEMENTARY FIGURE S7

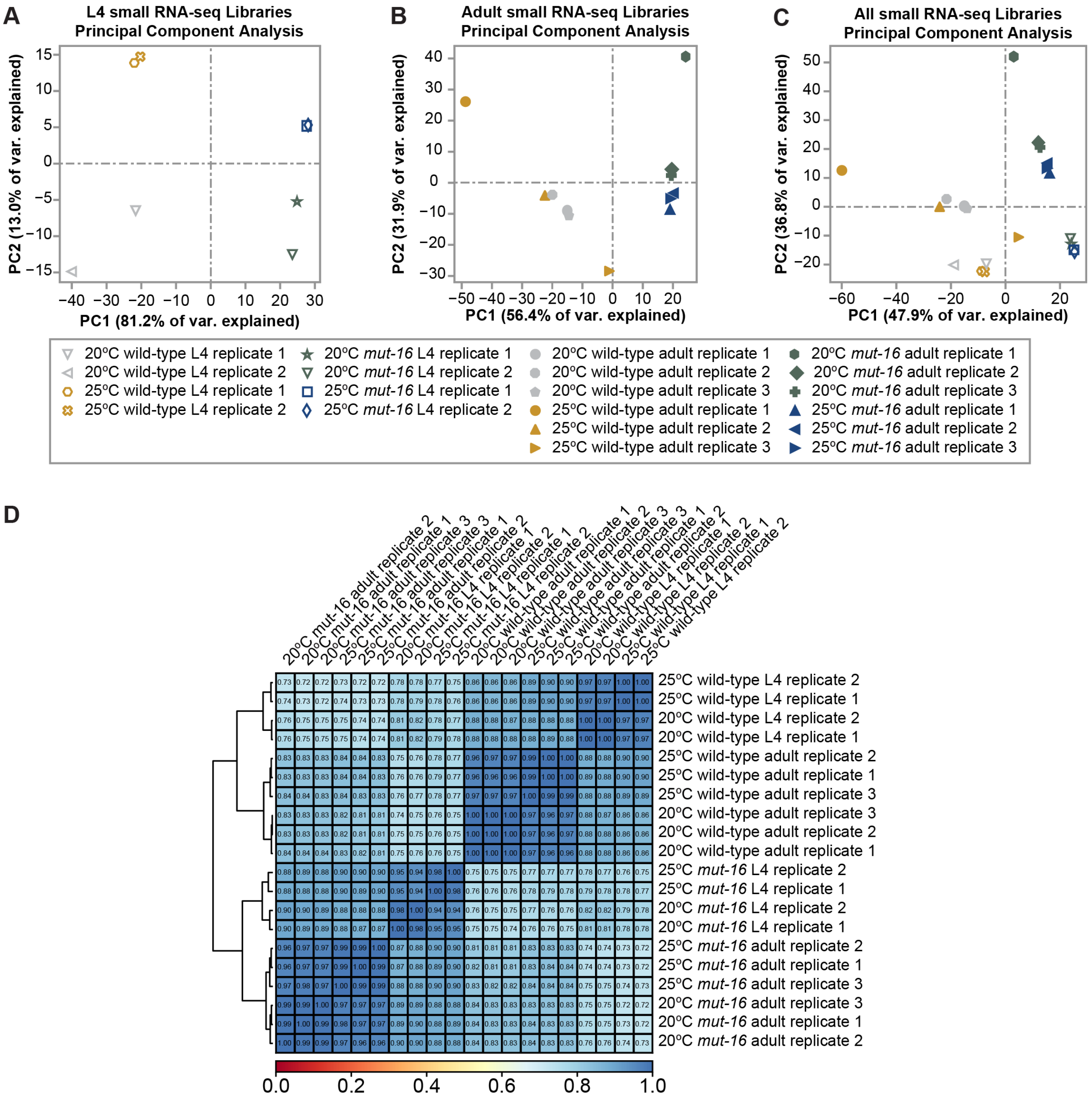


**Supplementary Figure S7.** Principal Component Analysis plots for **(A)** L4 small RNA-seq libraries, **(B)** adult small RNA-seq libraries, and **(C)** all small RNA-seq libraries. The key for the Principal Component Analysis plot symbols is shown below the plots. **(D)** Hierarchical clustering of global correlation coefficients between samples heat map for all small RNA-seq libraries.

**SUPPLEMENTARY TABLES.**

**Supplementary Table 1.** Library mapping statistics.

| **Library** | **Total Reads** | **Reads mapping to the WS258 genome** | **Reads mapping to WS258 mRNA, ncRNA, and pseudogenic transcripts** |
|---|---|---|---|
| N2 20ºC (L4) mRNA replicate 1 | 31,939,154 | 31,205,387 | 12,178,652 |
| N2 20ºC (L4) mRNA replicate 2 | 30,602,733 | 29,929,582 | 10,813,181 |
| *mut-16(pk710)* 20ºC (L4) mRNA replicate 1 | 24,731,385 | 24,088,431 | 9,914,053 |
| *mut-16(pk710)* 20ºC (L4) mRNA replicate 2 | 30,619,535 | 29,908,181 | 12,552,697 |
| N2 25ºC (L4) mRNA replicate 1 | 33,666,511 | 32,906,790 | 10,997,326 |
| N2 25ºC (L4) mRNA replicate 2 | 35,045,891 | 34,206,941 | 12,259,241 |
| *mut-16(pk710)* 25ºC (L4) mRNA replicate 1 | 33,728,292 | 32,980,606 | 12,276,552 |
| *mut-16(pk710)* 25ºC (L4) mRNA replicate 2 | 33,333,928 | 32,621,587 | 13,227,929 |
| N2 20ºC (L4) total small RNA replicate 1 | 15,614,472 | 13,455,532 | 5,028,484 |
| N2 20ºC (L4) total small RNA replicate 2 | 20,125,845 | 17,400,105 | 6,766,449 |
| *mut-16(pk710)* 20ºC (L4) total small RNA replicate 1 | 12,469,506 | 10,616,417 | 1,999,811 |
| *mut-16(pk710)* 20ºC (L4) total small RNA replicate 2 | 16,087,139 | 13,679,596 | 2,687,301 |
| N2 25ºC (L4) total small RNA replicate 1 | 15,716,559 | 13,559,035 | 4,826,359 |
| N2 25ºC (L4) total small RNA replicate 2 | 14,611,216 | 12,635,423 | 4,540,738 |
| *mut-16(pk710)* 25ºC (L4) total small RNA replicate 1 | 12,332,587 | 10,427,740 | 2,140,943 |
| *mut-16(pk710)* 25ºC (L4) total small RNA replicate 2 | 12,592,284 | 10,981,746 | 2,076,206 |

**Supplementary Table 2.** Oligonucleotide sequences.

| **Primer name** | **Sequence** |
|---|---|
| AR.020 *rpl-32* qPCR F | CAAGGTCGTCAAGAAGAAGC |
| AR.021 *rpl-32* qPCR R | GGCTACACGACGGTATCTGT |
| AR.030 *rab-3* qPCR F | GCCTTCGTCTCTACTGTCGG |
| AR.031 *rab-3* qPCR R | CGGCGGTATCCCAGATTTGA |
| AR.032 *myo-3* qPCR F | GCCTACGCTGATGCTCAGAA |
| AR.033 *myo-3* qPCR R | CCTTCTGGCGTTGTTCCTCT |
| AR.036 *faah-1* qPCR F | TTCACACCAACACCTGCACT |
| AR.037 *faah-1* qPCR R | TGGAATGACTGTATGTCCGGC |
| AR.038 F57F41.1 qPCR F | GGCCGCATCAATTTCAGACC |
| AR.039 F57F41.1 qPCR R | CGTGTTAACTCGGGCCTCTT |
| AR.050 *alg-3* qPCR F | GGATCTGGTTCACTGTCACC |
| AR.051 *alg-3* qPCR R | CGACAGGAGGTGATAAAGATCC |
| AR.052 *alg-4* qPCR F | CCTGCCACTTGCCGTAGTTA |
| AR.053 *alg-4* qPCR R | CCTTCGATCTTCCGTACTTCAC |
